# Pathogen-origin horizontally transferred genes contribute to the evolution of Lepidopteran insects

**DOI:** 10.1186/1471-2148-11-356

**Published:** 2011-12-12

**Authors:** Zi-Wen Li, Yi-Hong Shen, Zhong-Huai Xiang, Ze Zhang

**Affiliations:** 1 The Key Sericultural Laboratory of Agricultural Ministry, Southwest University, Chongqing 400715, China; 2 The Institute of Agricultural and Life Sciences, Chongqing University, Chongqing 400044, China

**Keywords:** Horizontal gene transfer, Insect evolution, Lepidoptera evolution, Functional innovation, Pathogenic bacteria

## Abstract

**Background:**

Horizontal gene transfer (HGT), a source of genetic variation, is generally considered to facilitate hosts' adaptability to environments. However, convincing evidence supporting the significant contribution of the transferred genes to the evolution of metazoan recipients is rare.

**Results:**

In this study, based on sequence data accumulated to date, we used a unified method consisting of similarity search and phylogenetic analysis to detect horizontally transferred genes (HTGs) between prokaryotes and five insect species including *Drosophila melanogaster*, *Anopheles gambiae*, *Bombyx mori*, *Tribolium castaneum *and *Apis mellifera*. Unexpectedly, the candidate HTGs were not detected in *D. melanogaster*, *An. gambiae *and *T. castaneum*, and 79 genes in *Ap. mellifera *sieved by the same method were considered as contamination based on other information. Consequently, 14 types of 22 HTGs were detected only in the silkworm. Additionally, 13 types of the detected silkworm HTGs share homologous sequences in species of other Lepidopteran superfamilies, suggesting that the majority of these HTGs were derived from ancient transfer events before the radiation of Ditrysia clade. On the basis of phylogenetic topologies and BLAST search results, donor bacteria of these genes were inferred, respectively. At least half of the predicted donor organisms may be entomopathogenic bacteria. The predicted biochemical functions of these genes include four categories: glycosyl hydrolase family, oxidoreductase family, amino acid metabolism, and others.

**Conclusions:**

The products of HTGs detected in this study may take part in comprehensive physiological metabolism. These genes potentially contributed to functional innovation and adaptability of Lepidopteran hosts in their ancient lineages associated with the diversification of angiosperms. Importantly, our results imply that pathogens may be advantageous to the subsistence and prosperity of hosts through effective HGT events at a large evolutionary scale.

## Background

Horizontal gene transfer (HGT) is a process in which exogenic DNA is introduced and integrated into a recipient genome. Any fraction of genetic materials can be transferred in general, but in fact, most persistently fixed sequences are transposable elements, functional genes and some regulatory sequences [1-4]. Transferred genes, as a type of genetic change at the level of "harbour or not", increase divergence between HGT recipients and their closely related species, which may result in innovations or improvements to physiological metabolism and other phenotypes of the hosts [5-8]. HGT is ubiquitous and abundant among prokaryotic organisms, and it is a major source of genetic variation in prokaryotes [9,10]. Making use of foreign genetic materials, microorganisms acquire novel functions to promote their fitness to particular niches [9,11]. Thus, HGT events among bacteria have biological significance for the evolution of prokaryotic organisms [11-13]. Compared with transfer frequency and amount in bacteria, HGT events among eukaryotes and between prokaryotes and eukaryotes are rare, especially for multicellular eukaryotes. This is partly attributed to the development of nuclear membrane and predominance of sexual reproduction in eukaryotic organisms [14-17]. Studies of HGT related to multicellular eukaryotes are not as prevalent as that among prokaryotes and unicellular eukaryotes. One reason is that contribution of HGT to the evolution of metazoan recipients may be small because of its rareness in multicellular eukaryotes. Nevertheless, case studies on HGT revealed that some of the transferred genes effectively participated in the biochemical metabolism and phenotypic divergence of multicellular eukaryotic hosts, implying that HGT may also have biological importance in the functional evolution of metazoan recipients [8,18-20]. Convincing evidence supporting this issue is still lacking.

HGT involved in insects and nematodes has been intensively investigated [17]. Based on population size and metabolic diversity, prokaryotes are considered as the major donor organisms for eukaryotic recipients [21]. Indeed, the majority of horizontally transferred genes (HTGs) have been found from endosymbiont bacterial species, *Wolbachia*, to their host insects [22-25]. A recent study also revealed that two pseudogenes in the aphid, *Acyrthosiphon pisum*, were horizontally transferred from *Buchnera aphidicola *(an aphid intracellular symbiotic bacterium) and four types of genes were obtained from other microorganisms [26]. Additionally, a fungal origin gene with function of carotenoid metabolism was found in the aphid genome [8]. In the silkworm, several HTGs were detected and their functions have been studied in detail [18-20,27]. A more recently study revealed 9 bacterial-origin HTGs in the silkworm genome, which was published just after submission of our manuscript [28]. These results indicated that insects have capability to integrate foreign genetic sequences into their genomes. In other words, HGT is also a way, seldom adopted but mostly efficient, by which insects can increase their genomic variation either from endosymbiont bacteria or from other microorganisms. Given that insects may be potential recipients of a relatively large amount of HTGs from microorganisms, a systematic study on insect HGT may help understand the contribution of HGT to the evolution of metazoan hosts.

Bioinformatics methods are commonly applied to detect candidate HGT events at genomics era [2,12,26,28,29]. The accumulated genome data of nearly 1000 bacteria and several insects make it possible to computationally detect HGT between microorganisms and insects at a genome level. Typical methods used for HGT detection in eukaryotes include homology search, analysis of sequence component and codon usage bias, distribution of homologous sequences, and phylogenetic analysis. These methods have different powers in revealing recent and ancient HGT events. In general, phylogenetic incongruity between a gene tree and the corresponding species tree is the most credible indicator of candidate transfer event in the detection of HGT [15,30]. However, sequence sampling bias and unsuitable tree-constructed methods may also result in incongruent topologies and false positives [14,21,30-32]. To avoid the false positives as far as possible, in this study, we not only perform a comprehensive homology search in public data to compensate for the sampling bias but also use three independent methods to reconstruct phylogenetic trees for each candidate HTG. In addition to taking efforts to improve the efficiency and accuracy of detection method, we employ a comparative strategy to detect HGT in the five insects with available genome sequences, *Drosophila melanogaster*, *Anopheles gambiae*, *Bombyx mori*, *Tribolium castaneum *and *Apis mellifera*, which belong to four different insect orders. Furthermore, the annotation information of their genomes is relatively abundant. We try to reveal the general features of bacteria-insect transferred genes such as transfer amount, transfer time, possible donor, evolutionary process and predicted functions. These general features will help understand the contribution and biological significance of foreign variations to the evolution of metazoan hosts.

## Methods

### Data collection

The predicted gene and protein sequence data of *D. melanogaster*, *Ap. mellifera*, *T. castaneum*, *An. gambiae*, and 994 prokaryotic organisms were downloaded from the National Center for Biotechnology Information (NCBI) web site (as of December 2009). The prokaryotic organisms include 926 eubacteria (22 classes, 621 species; Additional file 1) and 68 archaebacteria (5 classes, 59 species; Additional file 2). The predicted gene and protein sequences of the silkworm, *B. mori*, were obtained from Silkworm Genome Database (SilkDB) web site (as of December 2009) [33]. The predicted gene sets of 142 eukaryotic organisms with genome sequences available, including protista, fungi, plants and other animals, as well as some insects, were all downloaded from Kyoto Encyclopedia of Genes and Genomes (KEGG) web site (as of March 2010; Additional file 3). The species taxonomic information was obtained form NCBI taxonomy data (as of March 2010).

### Similarity search

**Figure 1:**
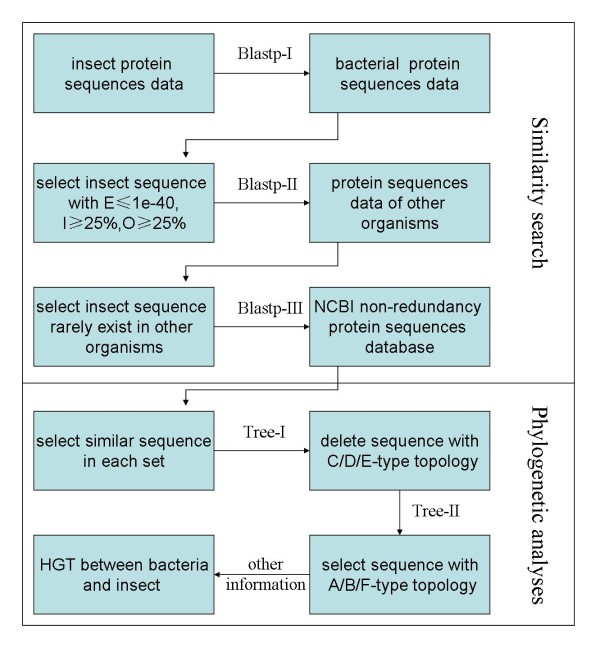
**Flowchart used in this detection**. In similarity search, E, O and I represent the E value, overlap value and identity value used in BLAST searches. In phylogenetic analysis, C/D/E-type topology represents non-HGT event and A/B/F-type topology represents HGT event, which are based on a previous study [30].

Identification of similar sequences between insects and prokaryotes is the initial step of HGT detection pipeline and it includes three steps (Figure 1).

The first step is blast-I. BLASTP search (BLAST 2.2.8) was performed to detect similar sequences between each insect (*D. melanogaster*, *An. gambiae, B. mori*, *T. castaneum*, *and Ap. mellifera*) and 994 bacteria with E value ≤ 10^-40^, overlap value ≥ 25% and identity value ≥ 25% [34]. The initial bacteria-insect similar amino acid sequence data were identified (Table 1).

**Table 1:** Numbers of remaining sequences after each procedure.

	*D. melanogaster*	*An. gambiae*	*B. mori*	*T. castaneum*	*Ap. mellifera*
Amino acid sequences used	20590	12659	14623	9833	9257
After blastp-I	2251	1337	1176	1493	1222
After blastp-II	179	154	163	154	183
After phylogenetic analysis	0	0	22	0	79

The second step is blast-II. Genome-wide predicted protein sequence data of other eukaryotic species with available genome sequences were separated into 6 sets: protista (29 species), fungi (48 species), plants (13 species), insects (22 species), non-insect arthropods (8 species), and non-arthropods metazoan (22 species). Using obtained bacteria-insect similar sequences as queries and BLASTP program with the same thresholds used in blast-I, we estimated the distribution spectrum of bacteria-insect similar sequences in the 6 sets of species. If there were more than two species in either of the 5 species sets (except for insect set) showing higher score and identity value than the corresponding top bacteria hit in blast-I, the insect sequence was deleted from candidate gene set because of its possible close relationship to gene in other eukaryotic species rather than bacteria. Insect sequences with no similar gene in other insects were recovered as candidate sequences.

The third step is using blast-III to search for similar sequences in species that their genome sequences are not available. We performed online BLASTP searches with the candidate genes querying the NCBI nonredundant protein sequence data which was separated into 7 species groups (eubacteria, archaebacteria, fungi, plants, arthropods, non-arthropod metazoans, and others). The thresholds used were E value ≤ 10^-3^, overlap value ≥ 25% and identity value ≥ 25%. Hit sequences containing the same protein domain(s) as the insect protein sequence were also selected as similar sequence when identity value ≤ 25%. The domain information was obtained from the NCBI DTT database.

### Phylogenetic analysis

Insect candidate HTGs and their similar sequences were used to construct phylogenetic trees, and judging standard of HGT or non-HGT event was based on a previous study in which phylogenetic topology patterns were particularly divided into 6 types to distinguish HTGs and others [30]. There are two steps in this section.

The first step is tree-I in which the phylogenetic trees were constructed using sequences obtained in blast-III. Similar sequences of each insect candidate gene were aligned using MUSCLE 3.6 [35], then the pairwise distance-matrix of aligned protein sequences was computed using PROTDIST program in PHYLIP 3.6 software package [36], and finally BIONJ [37] was employed to construct neighbor-joining (NJ) tree with the distance-matrix. We checked the topology of each candidate insect gene by hand using previous standard [30]. Insect genes with obvious topologies of vertical gene transfer were deleted. Remaining candidates, including genes with phylogenetic tree of HGT type as well as genes with complicated and disordered topologies in the simple NJ trees, were prepared for further analysis.

The second step is tree-II. We performed a detailed phylogenetic analysis by hand based on genes selected in tree-I. For the remaining candidate sequences, CLUSTALX 1.8 and MEGA 4.0 were used for NJ tree reconstruction [38,39], and 1000 bootstrap replicates were performed. For remaining genes with complicated topologies in tree-I, we selected similar sequences or sequence fragments for phylogenetic reconstruction with the same method above. After tree-II, insect genes with explicit topologies of HGT type were considered as the candidate sequences (Table 1).

### Determination of HGT events

Phylogenetic analyses in tree-I and tree-II were based on amino acid sequences of online available data. In addition, we used the detected insect candidate HTGs to query the NCBI nonredundant nucleotide sequences data to check for other species containing similar sequences of these HTGs, which might be missed in blast-III. The genomic contaminant sequences are one major cause of false positive in HGT detection. Information of GC content, intron number, chromosome location, EST (expressed sequence tag) sequence and expression information of the candidate HTGs were all analyzed to determine whether the detected candidates are contaminations or genes in the insect genomes. Thus, the candidate sequences through all the analyses above are considered as HTGs between bacteria and insect.

### Detecting direction and donor of HGT event

We used three methods, NJ, Bayesian inference (BI), and maximum likelihood (ML), to construct more refined phylogenetic trees of the detected candidates for the purpose of inferring the directions and possible donors of HGT events. For each candidate, we selected representative homologous sequences from species sets of bacteria, protista, fungi, plants and metazoan based on NJ trees constructed in tree-I and tree-II. Their protein domain regions were predicted in Pfam online services subsequently. Multiple alignments of the reduced amino acid sequences with domain regions were performed in CLUSTALX 1.8 and MUSCLE 3.6, and the results were checked by vision. ProtTest 2.4 was used to select a substitution model for tree construction for each HTGs at first [40]. WAG+gamma+Inv model was selected for all the HTGs. The Bayesian inference trees were constructed using MRBAYES 3.1.2 with WAG+invgamma model and 20000-1500000 generations were performed until the values of average standard deviation of split frequencies were stably below 0.01, then proper proportions of "burnin generations" were deleted to acquire topologies [41]. PhyML 3.0 was performed to construct the maximum likelihood (ML) trees and model of WAG+gamma+Inv was used [42]. MEGA 4.0 was used to construct NJ trees with JTT+gamma model and 1000 bootstrap resampling replicates [39]. Finally, topologies of BI trees were displayed in the result figures including support values displayed in ML and NJ trees. In the silkworm, a bacterial species for which a HTG clustered with its homolog can be considered as the corresponding candidate donor clearly. If there are a set of bacteria for which a HTG clustered with their homologs, then, the bacterium that lists in top of their BLAST hits is considered as the donor.

## Results

### HGT events from bacteria were detected only in the silkworm

With the pipeline of similarity search and phylogenetic analysis, we found 22 silkworm genes and 79 honeybee genes that are the candidate HTGs between insects and bacteria (Table 1). Unexpectedly, no candidate was detected in the fly, mosquito, and beetle in this study (Table 1). In the honeybee, none of the 79 genes has a corresponding EST sequence based on public sequence data, chromosomal location information, a neighboring gene in the sequencing fragment or a homologous sequence in the genome of wasp (another Hymenoptera insect) [43]. Additionally, the amino acid sequence identities between these genes and their top BLAST hits in bacteria are high (the average value is 69.5% with the highest 96.1% and the lowest 47.1%). The above features prompt us to doubt the existence of the 79 genes in the honeybee genome. Based on available sequence data, the most acceptable explanation for this question is that these 79 sequences may be genomic contaminations primarily coming from prokaryotes. However, 44 of these 79 sequences were predicted as the honeybee genes in the official gene set (OGS) downloaded from BeeBase in version of release 2 [44]. These genes were listed in Additional file 4 and were not included in the following analyses because of their uncertainty of genetic origin.

**Figure 2:**
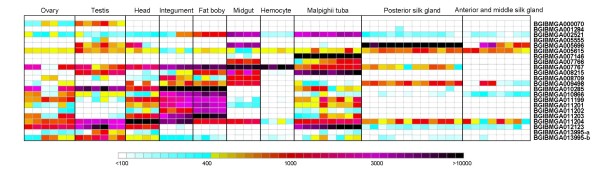
**Expression patterns of silkworm HTGs based on microarray signal intensities**. The color ruler is according to that in BmMDB web site. Numbers under it represent values of signal intensities. BGIBMGA013995 harbor two probes in data and they are displayed as -a and -b in this figure.

**Figure 3:**
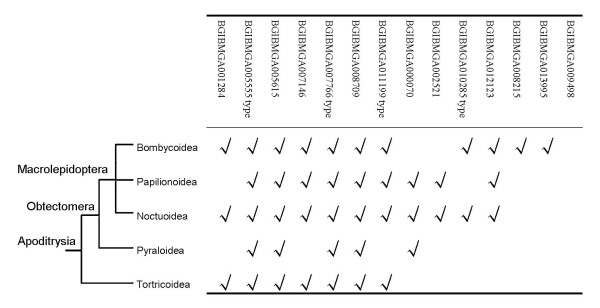
**Distribution of homologs of detected HTGs in Lepidoptera**. Phylogenetic tree indicates the general relationship of these five superfamilies in Lepidoptera, which is according to a previous scenario [77]. A detailed pattern contains species names and accession numbers is displayed in Additional file 5.

In the silkworm, 14 types of 22 genes were detected as the candidate HTGs (Table 2). The phrase "type of gene" refers to a transferred event, because a transferred gene may or may not duplicate in the recipients genome after its transfer. All previously revealed silkworm HTGs including 9 bacterial-origin HT genes in Zhu *et al*.'s result are included in our results [18-20,27,28]. Twenty one candidates were mapped in the silkworm chromosomes by SilkMap (a tool in SilkDB) [33], except for BGIBMGA005696. Ten of 22 candidates have EST evidence while 4 candidates (BGIBMGA005555, BGIBMGA005696, BGIBMGA007146 and BGIBMGA008709) were cloned in individual studies. In addition, 21 genes have expression information in the *B. mori *Microarray Database (BmMDB) [45] except for BGIBMGA00011200 (Table 2, Figure 2). Furthermore, 13 types of candidates have homologous sequences in other Lepidopteran insects except for BGIBMGA009498 (Figure 3, Additional file 5). EST fragments of 4 types of genes or their homologs in Additional file 5 (BGIBMGA002521, BGIBMGA005615, BGIBMGA010285 type, BGIBMGA012123 type) include poly-A tail structure. These results suggested that the 22 candidate HTGs detected in this study do exist in the silkworm genome and almost all of them are transcribed.

**Table 2:** Predicted prokaryote-origin HTGs in the silkworm

Gene ID	Annotation	Protein	GC	EST	Probe	Location		Top BLAST hit in bacteria			
						
		length	content			Chromosome	Interval	Species	Score	E	Identity (%)
BGIBMGA000070	yolk protein 1	267	0.36	0	sw05607	24	nscaf1108:2533757..2534560 (-)	*Methanosarcina barkeri *fusaro	446	1E-45	37.6
BGIBMGA001284	unkwn.	242	0.45	1	sw09798	13	nscaf1898:13442553..13443281 (+)	*Lactococcus lactis cremoris *MG1363	492	3E-51	41.3
BGIBMGA002521	γ-glutamyltranspeptidase	526	0.43	0	sw02154	9	nscaf2511:3929359..3930939 (+)	*Serratia proteamaculans *568	1642	0	59
BGIBMGA005555 BGIBMGA005696	β-fructofuranosidase (*BmSuc2*) β-fructofuranosidase (*BmSuc1*)	506 488	0.49 0.49	0 23	sw13105 sw02518	17 unkwn.	nscaf2829:2704806..2706326 (-) nscaf2830:395557..397023 (-)	*Bacillus amyloliquefaciens *FZB42 *Bacillus licheniformis *ATCC 14580	876 967	4E-95 1E-105	39 45.6
BGIBMGA005615	Zinc-type alcohol dehydrogenase-like protein	336*	0.42	3	sw13511	17	nscaf2829:935343..936353 (+)	*Alicyclobacillus acidocaldarius *DSM 446	478	2E-49	52.1
BGIBMGA007146	kynureninase (*BmKynu*)	426	0.42	1	sw14459	21	nscaf2868:1232908..1234188 (-)	*Alkaliphilus oremlandii *OhILAs	1237	1E-137	54.5
BGIBMGA007766 BGIBMGA007767	glycerophosphoryl diester phosphodiesterase glycerophosphoryl diester phosphodiesterase	378 372*	0.45 0.44	1 9	sw04248 sw16854	15 15	nscaf2888:43197..44333 (+) nscaf2888:49514..50632 (+)	*Pseudomonas aeruginosa* *Pseudomonas aeruginosa*	1252 1145	1E-138 1E-126	67.6 67.1
BGIBMGA008215	N-methyltryptophan oxidase	369	0.49	2	sw06048	18	nscaf2899:584306..585415 (-)	*Serratia proteamaculans *568	919	1E-100	48.6
BGIBMGA008709	chitinase (*BmChi-h*)	551	0.52	25	sw08485	7	nscaf2912:405540..407195 (+)	*Serratia proteamaculans *568	2100	0	72.9
BGIBMGA009498	ankyrin repeat domain protein	1632	0.38	0	sw09000	14	nscaf2953:1379654..1384552 (+)	*Wolbachia *endosymbiont of *Culex quinquefasciatus *Pel	1312	1E-145	33.2
BGIBMGA010285 BGIBMGA010866	NAD-dependent epimerase/dehydratase NAD-dependent epimerase/dehydratase	318 322	0.48 0.45	2 2	sw10878 sw09038	7 22	nscaf2986:5498917..5499873 (+) nscaf3005:1015921..1016889 (-)	*Photorhabdus asymbiotica* *Photorhabdus asymbiotica*	906 935	9E-99 1E-102	53.8 57.9
BGIBMGA011199 BGIBMGA011200 BGIBMGA011201 BGIBMGA011202 BGIBMGA011203 BGIBMGA011204	glucose-1-phosphatase glucose-1-phosphatase glucose-1-phosphatase glucose-1-phosphatase glucose-1-phosphatase glucose-1-phosphatase	391* 187 391* 391* 391* 394*	0.40 0.39 0.39 0.38 0.38 0.38	1 0 0 0 1 1	sw22572 unkwn. sw22571 sw22728 sw22676 sw06503	23 23 23 23 23 23	nscaf3026:4939042..49400217 (-) nscaf3026:4936929..4937492 (-) nscaf3026:4933133..4934308 (-) nscaf3026:4926408..49273583 (-) nscaf3026:4907767..4908942 (-) nscaf3026:4903958..4905142 (-)	*Serratia proteamaculans *568 unkwn.** Serratia proteamaculans 568 *Serratia proteamaculans *568 *Serratia proteamaculans *568 *Serratia proteamaculans *568	701 unkwn. 678 676 673 783	6E-75 unkwn. 3E-72 5E-72 1E-71 2E-84	40.8 unkwn. 40.2 39 39.6 42.2
BGIBMGA012123	pyridoxal-5'-phosphate-dependent protein β subunit	325	0.48	0	sw06559	11	nscaf3034:3775486..3776463 (+)	*Methylobacterium radiotolerans *JCM 2831	738	4E-79	47.2
BGIBMGA013995	glycosyl hydrolase	1077	0.40	0	sw05614 sw12345	28	nscaf3099:3081904..3085134 (+)	*Enterococcus faecalis *V583	3149	0	52.8

*: Gene sequence was corrected with corresponding EST sequence. So the length of predicted protein sequence is changed.

**: This gene fragment was not detected in the primary BLAST search.

Each of the detected silkworm candidates is located within the bacterial cluster in respective phylogenetic tree, which is significant evidence of HGT (Additional file 6, Figure S1 to S11; trees of other 3 types of genes are not shown because their homologs are very few in number). Additionally, the average similarity between 14 types of candidates and their bacterial hits is 50.36% (s.d., 10.84%) which is significantly larger than the mean value (mean, 41.03%; s.d., 2.62%) of random sampling distribution (14 samples were extracted randomly from the 1176 sequence similarity values of bacteria-silkworm homologous genes identified in blast-I, then the mean value was estimated, 10,000 replications), and there are 7 averages larger than 50.36% in the 10,000 random sampling results (P < 0.0007; Additional file 7). This suggested that HTGs are more similar to their bacterial homologs than vertically transferred genes. All of the detected candidates are intron-free genes, which is a trace of the bacteria-origin transferred genes. Simulations based on intron number of these 1176 genes (116 genes without intron) indicated that it is impossible to extract a group of intron-free genes (14 or 22 genes are randomly sampled each time) in 10,000 times of simulations (*P *< 0.0001). Thus, the detected 14 types of 22 genes should be HTGs between prokaryotes and silkworm.

Among the 14 types of detected silkworm HTGs, the functions of *BmSuc *(BGIBMGA005555 and BGIBMGA005696), *BmKynu *(BGIBMGA007146) and *BmChi-h *(BGIBMGA008709) were previously characterized [18-20]. And BGIBMGA011199 type may code the bacterial type glucose-1-phosphatase [46]. Additionally, BGIBMGA005615, BGIBMGA007766 type, BGIBMGA010285 type and BGIBMGA011199 type all have the conserved catalytic residues and/or cofactor bonding domains based on available crystal structure information of their bacterial homologs, except for BGIBMGA008215 whose substrate recognizing motifs were replaced (Additional file 6, Figure S3, S5, S6, S8 and S9) [47-52]. The remaining 6 types of the detected HTGs all have transcription evidence in BmMDB (Figure 2). Thus, the detected 14 types of silkworm HTGs are active genes in the host.

### The silkworm HTGs have homologs in other Lepidopteran insects

**Figure 4:**
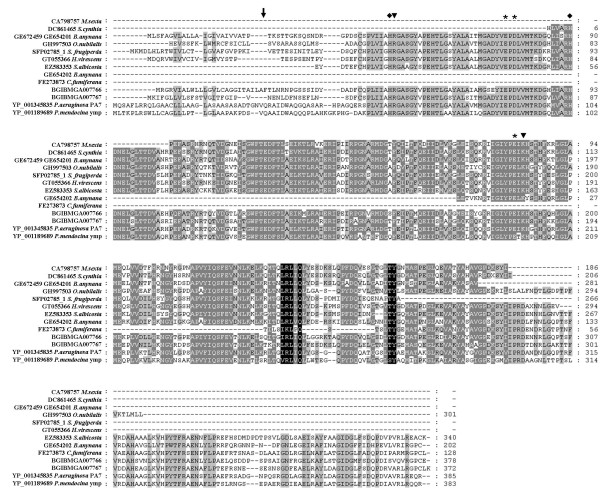
**Multiple alignment of amino acid sequences of BGIBMGA007766, BGIBMGA007767 and their homologs**. Arrow represents the predicted cleavage site of signal peptide. Asterisk represents metal-binding site. Rhombus represents essential residue for catalysis. Trigone represents other conserved site.

**Figure 5:**
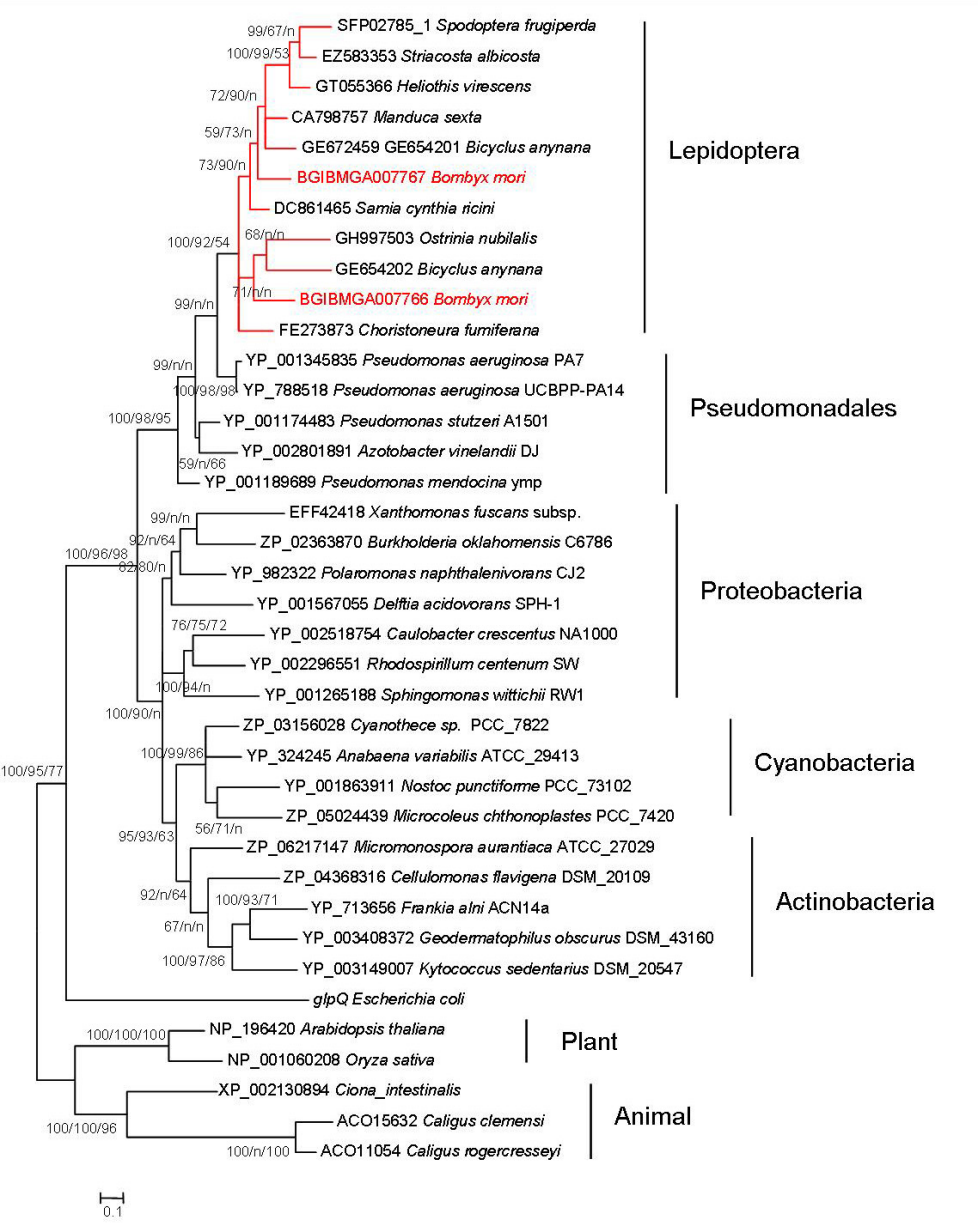
**Phylogenetic tree of BGIBMGA007766, BGIBMGA007767 and their homologs**. Numbers beside nodes indicate supporting values in methods of BI/ML/NJ.

Previous studies indicated *BmSuc *(BGIBMGA005555 and BGIBMGA05696) and *BmChi-h *(BGIBMGA008709) have homologous genes in other Lepidopteran insects [18,53]. Using the detected 14 types of silkworm HTGs as queries, we searched for their homologous sequences in other Lepidopteran insects (Figure 3, Additional file 5). Homologous sequences of each type of genes were used to construct phylogenetic trees with bacterial sequences in Additional file 6, respectively. The Lepidopteran sequences clustered with the silkworm HTGs as monophyletic group are considered as homologous sequences of the silkworm HTGs. We found that homologs of the detected HTGs are widely distributed in the Ditrysia insects rather than only in the silkworm except for BGIBMGA009498. Thus, the majority of them are not HTGs between bacteria and silkworm as previously thought, instead, they are bacterial genes fixed into the ancient Lepidopteran insect genomes (Figure 4 and 5). There are 4 hierarchies in Figure 3 based on the phylogenetic relationships of Lepidopteran superfamilies. The first group is BGIBMGA008215 and BGIBMGA013995, which have homologs only in Bombycoidea; the second group contains BGIBMGA002521 and BGIBMGA010285 type, of which the homologs exist in Macrolepidoptera (including Bombycoidea, Papilionoidea and Noctuoidea in Figure 3); the third group is BGIBMGA000070 detected in Obtectmera (including Macrolepidoptera and Pyraloidea); and the fourth group is related to Apoditrysia, including BGIBMGA001284, BGIBMGA005555 type, BGIBMGA005615, BGIBMGA007146, BGIBMGA007766 type, BGIBMGA008709 and BGIBMGA011199 type.

In fact, the distribution pattern of homologous sequences shown in Figure 3 not only depends on the real transfer time and evolutionary process of Lepidopteran HTGs, but also is affected by the extent and abundance degree of sequence data accumulated in public database. Most of sequences in Figure 3 are ESTs downloaded from NCBI and ButterflyBase [54], thus we analyzed the components of NCBI EST data based on the major taxonomical groups of Lepidoptera. Consequently, in NCBI, there are 309,472 EST sequences in silkworm, 70,920 in Bombycoidea (excluding the silkworm ESTs), 166,569 in Noctuoidea, 163,963 in Papilionoidea, 21,208 in Pyraloidea, 79,438 in Tortricoidea, and 920 in Tineoidea. In total, 814,135 ESTs of Lepidopteran insects have been accumulated in NCBI, 99.8% of which belong to Ditrysia group. And this ratio is consistent with that 98% of extant Lepidopteran insect species are ascribed to Ditrysia group [55]. It is clear from these data that sequence number in Pyraloidea or Tortricoidea is less than half of that in Noctuoidea or Papilionoidea, which may reduce the detected distribution range of genes in the second and third groups (BGIBMGA000070, BGIBMGA002521 and BGIBMGA010285) in Figure 3. The distribution of homologous sequences for an HTG provides primary evidence to roughly infer its transfer time, at least lower bounnd on the time. Considering the effect of less sequence data for Pyraloidea and Tortricoidea, the tendency in Figure 3 is that at least 10 types of genes (including genes in the second and third groups) may come from relatively distant HGT events which are traced back to the ancestor of Bombycoidea and Tortricoidea insects, and 3 types (BGIBMGA008215, BGIBMGA009498 and BGIBMGA013995) limited in Bombycoidea may come from relatively recent events. Thus, most of these genes may be introduced into Lepidoptera before radiation of Ditrysia or Apoditrysia group.

There is an exceptional case in the similarity search based on ESTs. BGIBMGA012123 contains homologs in Bombycoidea, Papilionoidea and Noctuoidea in Lepidopteran insects (Figure 3). Unexpectedly, three ESTs belonging to three different non-insect arthropods (*Folsomia candida*, EV479859; *Ixodes scapularis*, EW883321; *Tetranychus urticae*, GT984060) are similar to BGIBMGA012123. The corresponding phylogenetic analysis indicated that these three ESTs and BGIBMGA012123 formed a monophyletic group that clustered within bacteria. If these three ESTs are reliable, there may be gene loss events in other insect orders. However, there is another hypothesis that this type of gene would be acquired in ancestor of arthropods and it was lost in most of insect orders except for Lepidoptera. It is also possible that this gene was independently transferred into Lepidoptera and those arthropods, respectively. As a candidate HGT, this gene was also used in following analysis.

### The majority of the predicted donors are entomopathogenic bacteria

After constructing phylogenetic trees for 11 types of silkworm HTGs (Additional file 6, Figure S1 to S11), the donors of these genes were predicted (Table 3).

**Table 3:** Predicted prokaryotic donors of the detected Lepidopteran HTGs

Gene ID	Top BLAST hit	Phylogenetic tree	Predicted donor	Relationship	Phylum	Class	Order	Family
BGIBMGA002521	*Serratia proteamaculans *568	a set of bacteria	*Serratia *bacterium	insect pathogen	Proteobacteria	Gammaproteobacteria	Enterobacteriales	Enterobacteriaceae
BGIBMGA005555 BGIBMGA005696	*Bacillus amyloliquefaciens *FZB42 *Bacillus licheniformis *ATCC 14580	a set of bacteria	*Bacillus *bacterium	insect pathogen	Fimicutes	Bacilli	Bacillales	Bacillaceae
BGIBMGA005615	*Alicyclobacillus acidocaldarius *DSM 446	*Listeria grayi *DSM 20601	*Listeria *bacterium	-	Fimicutes	Bacilli	Bacillales	Listeriaceae
BGIBMGA007146	*Alkaliphilus oremlandii *OhILAs	*Listeria grayi*	*Listeria *bacterium	-	Fimicutes	Bacilli	Bacillales	Listeriaceae
BGIBMGA007766 BGIBMGA007767	*Pseudomonas aeruginosa*	*Pseudomonas aeruginosa *UCBPP-PA14	*Pseudomonas *bacterium	insect pathogen	Proteobacteria	Gammaproteobacteria	Pseudomonadales	Pseudomonadaceae
BGIBMGA008215	*Serratia proteamaculans *568	a set of bacteria	*Serratia *bacterium	insect pathogen	Proteobacteria	Gammaproteobacteria	Enterobacteriales	Enterobacteriaceae
BGIBMGA008709	*Serratia proteamaculans *568	a set of bacteria	*Serratia *bacterium	insect pathogen	Proteobacteria	Gammaproteobacteria	Enterobacteriales	Enterobacteriaceae
BGIBMGA009498	*Wolbachia *endosymbiont of *Culex quinquefasciatus *Pel	-	*Wolbachia *bacterium	insect symbiont	Proteobacteria	Alphaproteobacteria	Rickettsiales	Rickettsiaceae
BGIBMGA010285 BGIBMGA010866	*Photorhabdus asymbiotica*	*Photorhabdus asymbiotica* *Photorhabdus luminescens*	*Photorhabdus *bacterium	insect pathogen	Proteobacteria	Gammaproteobacteria	Enterobacteriales	Enterobacteriaceae
BGIBMGA011199 BGIBMGA011200 BGIBMGA011201 BGIBMGA011202 BGIBMGA011203 BGIBMGA011204	*Serratia proteamaculans *568	*Aggregatibacter aphrophilus *nj8700	*Serratia *bacterium	insect pathogen	Proteobacteria	Gammaproteobacteria	Pasteurellales	Pasteurellaceae
BGIBMGA012123	*Methylobacterium radiotolerans* JCM 2831	*Methylobacterium radiotolerans *JCM 2831 *Sagittula stellata *E-37	*Methylobacterium *bacterium	plant symbiont	Proteobacteria	Alphaproteobacteria	Rhizobiales	Methylobacteriaceae
BGIBMGA013995	*Enterococcus faecalis *V583	*Enterococcus faecalis*	*Enterococcus *bacterium	insect pathogen	Fimicutes	Bacilli	Lactobacillales	Enterococcaceae

We found that there are 2 donor bacteria in class Alphaproteobacteria of phylum Proteobacteria, 6 donors in class Gammaproteobacteria of phylum Proteobacteria, and 4 belonging to class Bacilli of phylum Fimicutes (Table 3). Bacteria in four genera (Serratia, Photorhabdus, Pseudomonas and Bacillus) are the major source of pathogenic microorganisms which induce diseases of bacterial septicemia, toxinosis, and intestinal disease for Lepidopteran insects and other insects [56-61]. The virulent protein of *Enterococcus faecalis *V583 is a lethal protein to the Lepidopteran insect, indicating that it may be also an entomopathogenic bacterium [62,63]. BGIBMGA012123 clustered with *Methylobacterium radiotolerans *JCM 2831, which is also the top BLAST hit species, and most of *Methylobacterium *species are plant symbiotic bacteria [64,65].

The prokaryotes used in this study include nearly one thousand of organisms (994 organisms in 680 species), which is just a small sample of the prokaryotic population in biosphere. Thus, we analyzed the species components of the 994 bacterial genomes with the purpose of simply estimating the effect of the sample component on the donor inference. There are 315 species (46.3% of 680 species) of 486 organisms (48.9% of 994 organisms) in phylum Proteobacteria and 99 species (14.6%) of 184 organisms (18.5%) in phylum Fimicutes, which constitute two primary parts of bacterial organisms used. In the data, class Gammaproteobacteria contains 125 species (18.4%) of 236 organisms (23.7%) and class Bacilli contains 63 species (9.3%) of 133 organisms (13.4%), and the proportions of them are still large. It is known that Proteobacteria is the largest phylum in bacteria (more than 40% published bacterial genera belong to it), and Gammaproteobacteria is the largest class in Proteobacteria. Generally, species component of 994 genomes used in this study corresponds to the real component of bacteria phylum in some extent. Therefore, donor results at phylum and class level are normal and reasonable. At the genus level, only 3.4% of bacterial species used in this study are common entomopathogenic bacteria, including genera of *Serratia *(1 species), *Photorhabdus *(2 species), *Pseudomonas *(9 species) and *Bacillus *(11 species). Additionally, 55 (4.7%) of these 1176 silkworm genes detected after blast-I have homologs in above four bacterial genera based on their top BLAST hits. However, a half (BGIBMGA002521, BGIBMGA005555 type, BGIBMGA007766 type, BGIBMGA008215, BGIBMGA008709, BGIBMGA010285 type and BGIBMGA011199 type) of HTGs are entomopathogenic bacterial origin. Thus, these observations imply that insect pathogenic bacteria were the major donors of Lepidopteran HTGs.

## Discussion

### Uneven transfer amount from bacteria to insects

Although the five available insect genomes were analyzed for HGT, significant HGT events were detected only in the silkworm. Three reasons may explain this observation. The first is utilization of incomplete sequence data in this study. In general, before the assembly of a genome sequence, the reads are checked to get rid of contaminant fraction from symbiont, parasite and pathogen. As a result, HTG sequences coming from other organisms may be deleted as contaminants. The second is that the power of detection methods used in this study is not high. Similarity search and phylogenetic analyses and other information were combined as a detection pipeline to reveal HGT event; this reduces the ratio of false positives. However, the complex steps and strict standards in the pipeline may also reduce the number of the detected candidates. The third is that the contrast in HTG numbers between the silkworm and other four insects may be true.

*Wolbachia *species are endosymbionts infecting 20% of arthropods on Earth [66,67], and the genetic fractions of this prokaryote were detected in the genomes of numerous insects [22,23,68,69]. This suggests that these insects have ability to accept foreign genetic materials. Additionally, the *D. melanogaster *genome was first sequenced among the five insects, and its annotation is more complete and detailed. However, the trail of HTG was not found in *D. melanogaster *based on its sequence data. Consequently, incomplete data is not a reasonable explanation. Various types and large amount of Lepidopteran HTGs do exist. In addition, we found 79 suspected sequences in *Ap. mellifera*, which are considered as contaminants. Strikingly, all previously reported silkworm transferred genes were recovered in this study [18-20,27], especially, a recent and independent study on the silkworm HGT also confirmed the methods and results in our study [28]. Thus, the detection pipeline we used appears to be powerful and should not miss real HTGs. In conclusion, HGT events may be distributed unevenly at least in four major insect orders of the five insects. Silkworm (Lepidoptera) is a distinct case in which HTGs are common and functional. The disparity of the transfer number and ratio of HTGs in a certain organism category has been shown previously in an HGT study on fungi [29].

The proportion (0.15%) of the transferred genes in silkworm genome is comparable with the average level (0.12%) in fungi [29]. In chromalveolates, a group of protista, 16 types of bacteria transferred genes were detected [70]. However, as simple eukaryotes, fungi and protista are thought to accept foreign genetic sequences into their genomes more easily. In aphid, 6 types of 12 genes including 3 pseudogenes were detected as HTGs and its proportion ranges from 0.11% to 0.03% (0.08% to 0.03% when deleting pseudogenes) [26,71]. Thus, the amount of Lepidopteran HTGs is relative large. Whether some ancient Lepidopteran insects have an unusual ability of acquiring and fixing foreign genetic materials is unclear. A previous study on prokaryotes suggested that the proportion of distant HTGs is correlated to the genome sizes of donors and recipients [72]. Therefore, a large proportion of HTGs in Lepidopteran insects may be, in part, attributed to the genome structure and component (e.g., genome size, transposable element, recombination rate, metabolic network and so on) of these recipients. However, difference in selection pressure among insects due to their surrounding niches is another determinant and may result in uneven transfer amount among bacteria and insects.

### The evolutionary characteristics of Lepidopteran HTGs

There are common features of HGT in prokaryotes and eukaryotes. In prokaryotes, the sequence composition of anciently transferred genes are often ameliorated to the host genome [73]. As expected, GC contents of the transferred genes in silkworm display a more centralized distribution (mean: 43.83%, s.d.: 4.82%) compared with that of the predicted bacterial donors (mean: 49.23%, s.d.: 11.27%; Additional file 8). This also indicates that most of these transferred genes have been integrated into recipient genome for a long period of time, which is consistent with the EST search results in Lepidoptera (Figure 3). Three of the 14 types of Lepidopteran HTGs (β-fructofuranosidase, glycerophosphoryl diester phosphodiesterase and NAD-dependent epimerase/dehydratase) contain multiple genes. The glucose-1-phosphatase consists of 6 tandem arranged genes (Table 2). However, these gene duplication events were not revealed in a recent study [28]. In aphid, the transferred gene of rare lipoprotein A (*RlpA*) has 5 duplicated genes which are also tandem arranged, and another gene type, LD-carboxypeptidase_1 (*LdcA*), includes 2 genes with one inactive [26]. At least more than a quarter of the detected HTGs were duplicated after HGT events, either in Lepidoptera or in aphid. This tendency is consistent with the findings in prokaryotes that the transferred genes are more frequently duplicated than endogenetic genes in hosts [74]. We also found that some detected HTGs (BGIBMGA002521, BGIBMGA007766 type, BGIBMGA008709) harbour respective homologs which are vertically transferred genes in the silkworm and other insect genomes; that is, these HTGs and their homologs belong to the same protein family. In a recent study, the significant contribution of HGT to the expansion of protein families in bacteria was revealed [13]. Thus, HGT events also affect the evolution of protein families in Lepidopteran insects, more or less.

There are some special aspects for the HTGs detected in this study. Potential prokaryotic donors of insects include symbionts, parasites, pathogens and bacteria in diet and surrounding environments. Previous studies on HGT between prokaryotes and insects mainly focused on the insect endosymbiont bacteria [17]. In this paper, we also found that one gene (BGIBMGA009498) might be transferred from *Wolbachia *bacterium. However, the experimental evidence indicated that extant silkworm may be not infected by *Wolbachia *species [75]. Similar results were found in *Aedes aegypti *and two filarial nematode species [24,76]. At least 7 types of HTGs may be introduced from entomopathogenic bacteria (pathogenic bacteria) and another donor is an endophytic bacterium (bacteria in food) (Table 3). Thus, donor pattern of Lepidopteran HTGs is multifarious, which is different from that in aphid and other insects. A recent HGT event can be detected more easily than ancient ones, because sequence similarity between donor and the transferred sequence will decrease and base composition of transferred sequence will ameliorate to the recipient genome after the fixation [15]. In aphid, *RlpA *gene was transferred from a relatively ancient HGT event about 50-70 MYA ago [26]. In HGT studies between insects and *Wolbachia*, the majority of genes were transferred from endosymbiont to hosts recently. Based on phylogenetic topologies and homolog distribution (Figure 3), we found that most of Lepidopteran HTGs might be integrated into the hosts at least before the radiation of Ditrysia or Apoditrysia group, about 100 MYA ago [77]. This indicates that the method we used is effective in revealing ancient HGT events. Additionally, these detected Lepidopteran HTGs, as a group of special-origin genetic fragments, can be used in the phylogenetic reconstruction of Lepidopteran insects, especially for Ditrysia insects among which the phylogenetic relationships are not clear in detail [55].

### The biological significance of Lepidopteran HTGs in the evolution of hosts

A question may arise: why do the transfer times of most detected HGT events in Lepidoptera fall within a relatively narrow evolutionary period? Lepidopteran insects are a relative young biological group in geology history compared with other insect orders. Furthermore, Lepidoptera are the second largest order in insecta and the largest group in plant-feeding insects. A generally accepted opinion holds that the prosperity of Lepidopteran insects is associated with the diversification of angiosperms on Earth in the late Cretaceous period which is just about 100 MYA ago [77]. At that time, developing angiosperms provide rich foods and living environments for some ancient Lepidopteran insects (may be the ancestor of Ditrysia insects). Thus, the genetic changes that facilitated hosts to adapt to the new niches predominated by angiosperm would be fixed in the ancient Lepidopteran lineages. Biological traits related to nutrition, reproduction, defense and immunization, are major targets of natural selection. Almost all of the detected HTGs are functional enzymes except for BGIBMGA000070 which may be a storage protein. Furthermore, previous studies revealed that one HTG BGIBMGA007146 in the silkworm participated in degradation, modification and combination of the toxins [20], and another HTG BGIBMGA011204 improved the metabolic pathway to get out of the toxic target site(s) [46]. Again, the silkworm HTG BGIBMGA005696 is involved in replacement of the targeted enzyme with a resistant one [19]. Thus, the majority of Lepidopteran HTGs might perform physiological functions in nutritional metabolism and detoxification. Detoxification is related to the nutritional metabolism because toxins in diet and toxins generated in normal endogenetic metabolisms can obstruct and reduce the ingestion and digestion activities of consumers. In a long interaction history between insects and their dietary plants, phytophagous insects might have developed some effective strategies to protect the efficiency of nutritional metabolism from the detriments produced by plants. The HTGs from bacteria may have contributed novel functions for Lepidopteran hosts to adapt to various diets and niches.

It is interesting that a half of the detected HTGs are pathogen-origin, while previous studies indicated that bacteria of endosymbionts and parasites may be the primary donor group of HGT. It is well known that obligate symbiotic and parasitic bacteria are often degenerated in some physiological metabolisms and phenotypic traits [78-80]. Thus, potential genetic variations they could offer for recipients may be not plentiful and effective to increase host's adaptability in complex niches, especially when surrounding environments shifted [81,82]. This is probably a reason that most of symbiont-origin HTGs in insects and nematodes are nonfunctional or inactivated [23,25,26]. In contrast, pathogenic bacteria in this study are more complex in ecological niche and biological functions compared with obligated symbiotic bacteria [56,58-60]. Previous studies revealed several HGT events from non-endosymbiosis organisms to multicellular recipients [9,16,26]. However, the mechanism of foreign sequences transferred into the recipient germline from non-endosymbiosis organisms is unclear. Thus, how these pathogen genes integrated into the Lepidopteran insects remains to be elucidated.

Whether HGT from other organisms to multicellular eukaryotes has biological significance in the evolutionary process of hosts is unknown. Two factors may affect the evolutionary significance of the transferred genes: their persistence in host genome and their integration in host biology [83]. On the basis of previous individual cases, some transferred genes certainly perform functions in the host. However, in this study, we found a relative large group of ancient transferred genes in Lepidopteran insects, and these genes are predicted to have biological functions since they were integrated into ancient Lepidopteran genomes. Furthermore, the integration of these HTGs into Lepidoptera at least corresponds to the expansion of angiosperm. Thus, it is most likely that most of these HTGs facilitated Lepidoptera to adapt to the evolution of their plant hosts. In short, our results provide new evidence to support for exogenic variations significantly contributing to the evolution of metazoan organisms.

## Conclusions

In this study, we applied a uniform method including sequence similarity, homolog distribution, phylogenetic incongruity and other information to detect HGT events between bacteria and the five insects. Unexpectedly, 14 types of 22 HTGs were detected only in the silkworm. Further study suggested that most of these HTGs are Lepidoptera specific. Moreover, the estimate of the transfer time of these HTGs into Lepidoptera corresponds to the evolutionary age of angiosperm expansion. Since most Lepidoptera are phytophagous insects and the majority of HTGs may perform physiological functions in nutritional metabolism and detoxification, these HTGs facilitated Lepidoptera to adapt to the evolution of their plant hosts. Thus, our results provide some insight into understanding the biological significance of HGT to the evolution of metazoan recipients.

## Authors' contributions

ZZ made the study design. ZWL carried out the analyses, and drafted the manuscript. YHS read the manuscript. ZZ revised the manuscript. ZZ and ZHX supervised the study. All authors read and approved the final manuscript.

## Authors' information

1. The Key Sericultural Laboratory of Agricultural Ministry, Southwest University, Chongqing, 400715, China. 2. The Institute of Agricultural and Life Sciences, Chongqing University, Chongqing 400044, China.

## Supplementary Material

Additional file 1 **Eubacterial organisms used in this detection**. Click here for file

Additional file 2 **Archebacterial organisms used in this detection**. Click here for file

Additional file 3 **Other organisms used in this detection**. Click here for file

Additional file 4 **Detected doubtful sequences in honeybee**. Click here for file

Additional file 5 **Homologous sequences of silkworm HTGs in other Lepidopteran insects**. Click here for file

Additional file 6 **Multiple alignment of amino acid sequences and phylogenetic trees of detected HTGs**. Click here for file

Additional file 7 **Average similarity between detected HTGs and predicted donor sequences**. Red curve indicates the normal distribution (mean, 41.03%; s.d., 2.62%) of sequence similarity based on random sampling results. Blue curve indicates the normal distribute (mean, 41.02%; s.d., 9.76%) of sequence similarity between 1176 silkworm genes and their bacterial homologs. Arrow indicates the position of mean value (50.36%) of 14 types of HTGs. Click here for file

Additional file 8 **GC content distribution of detected HTGs**. The normal distribution (mean, 47.87%; s.d., 7.86%) indicates GC contents of the silkworm 14,623 genes. The open circles represent GC contents of silkworm HTGs (from left to right: BGIBMGA000070, BGIBMGA009498, mean value of BGIBMGA011199 type, BGIBMGA013995, BGIBMGA005615, BGIBMGA007146, BGIBMGA002521, mean value of BGIBMGA007766 type, BGIBMGA001284, mean value of BGIBMGA010285 type, BGIBMGA012123, mean value of BGIBMGA005555 type, BGIBMGA008215, BGIBMGA008709). The solid circles represent GC contents of corresponding donor sequences. The donors and recipients are connected with arrow lines. Click here for file
