# Supplementary figures and images for: Pathogen-origin horizontally transferred genes contribute to the evolution of Lepidopteran insects
Source: BMC Evol Biol. 2011 Dec 12;11:356. doi: 10.1186/1471-2148-11-356 (PMC3252269; doi:10.1186/1471-2148-11-356)

**Additional file 1** Eubacterial organisms used in this detection


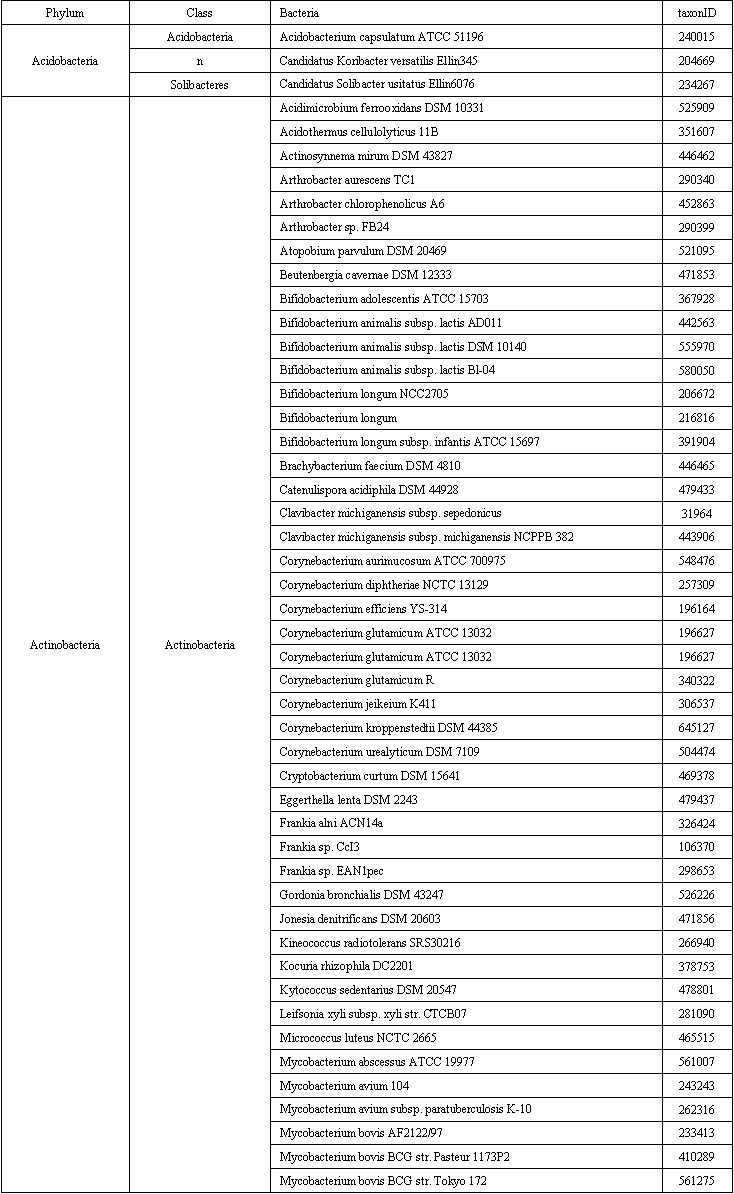


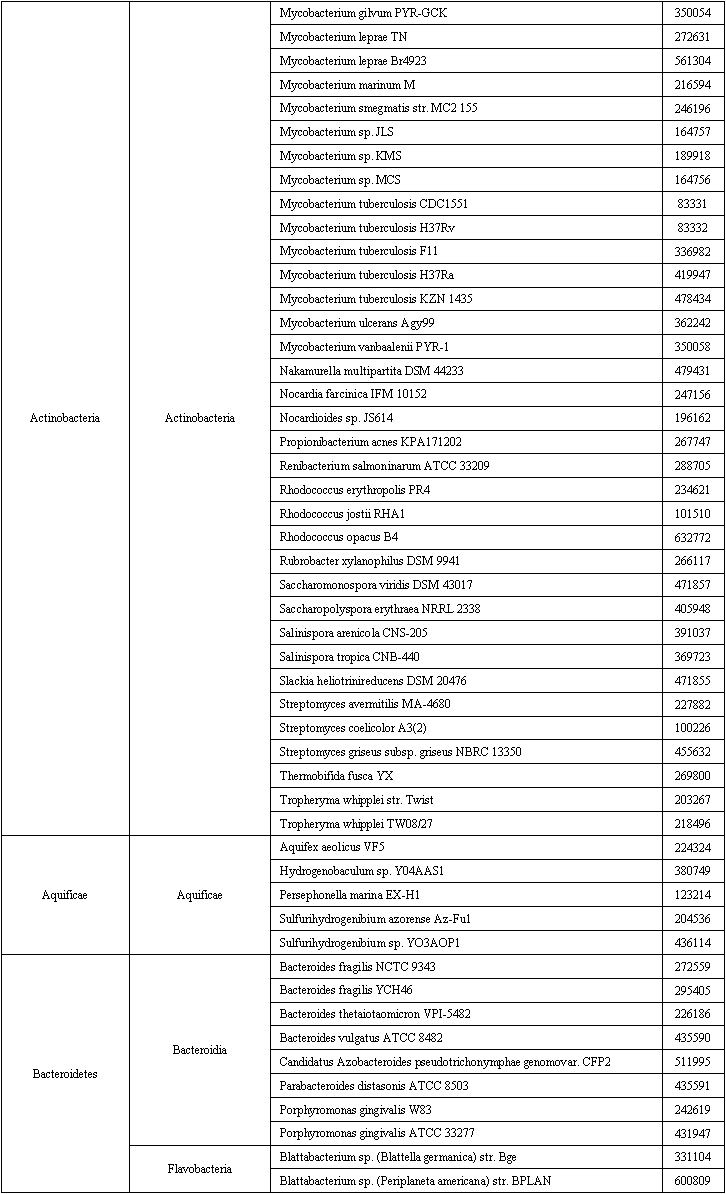


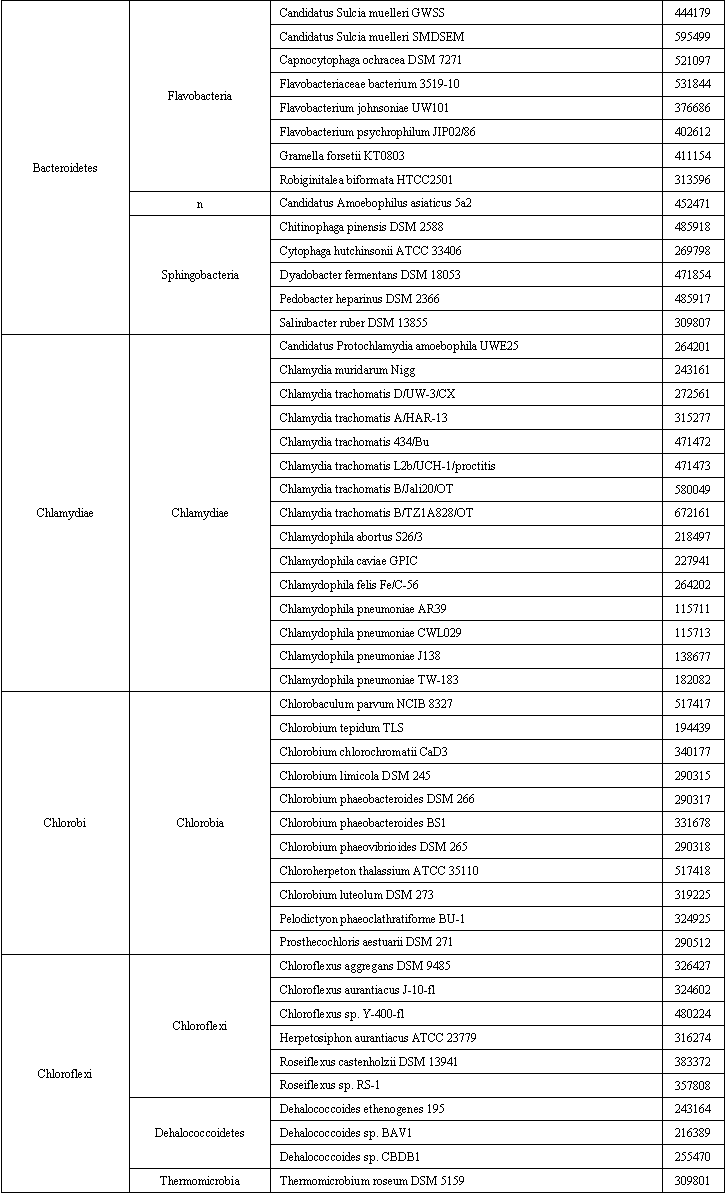


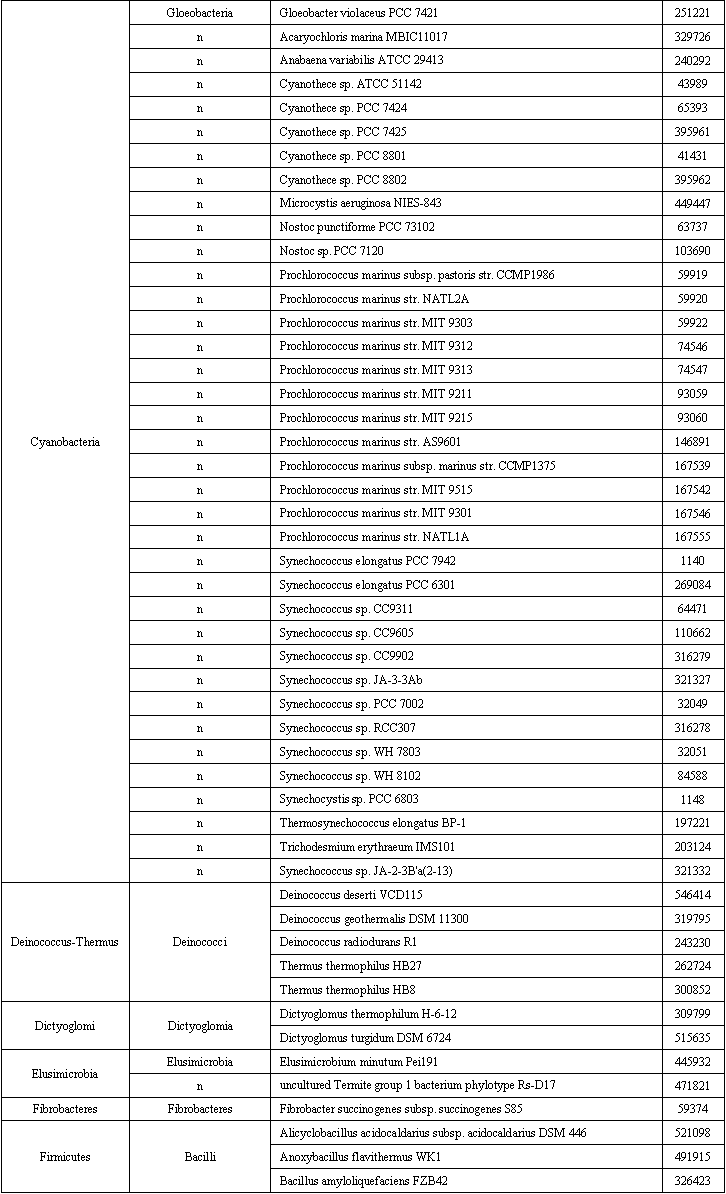


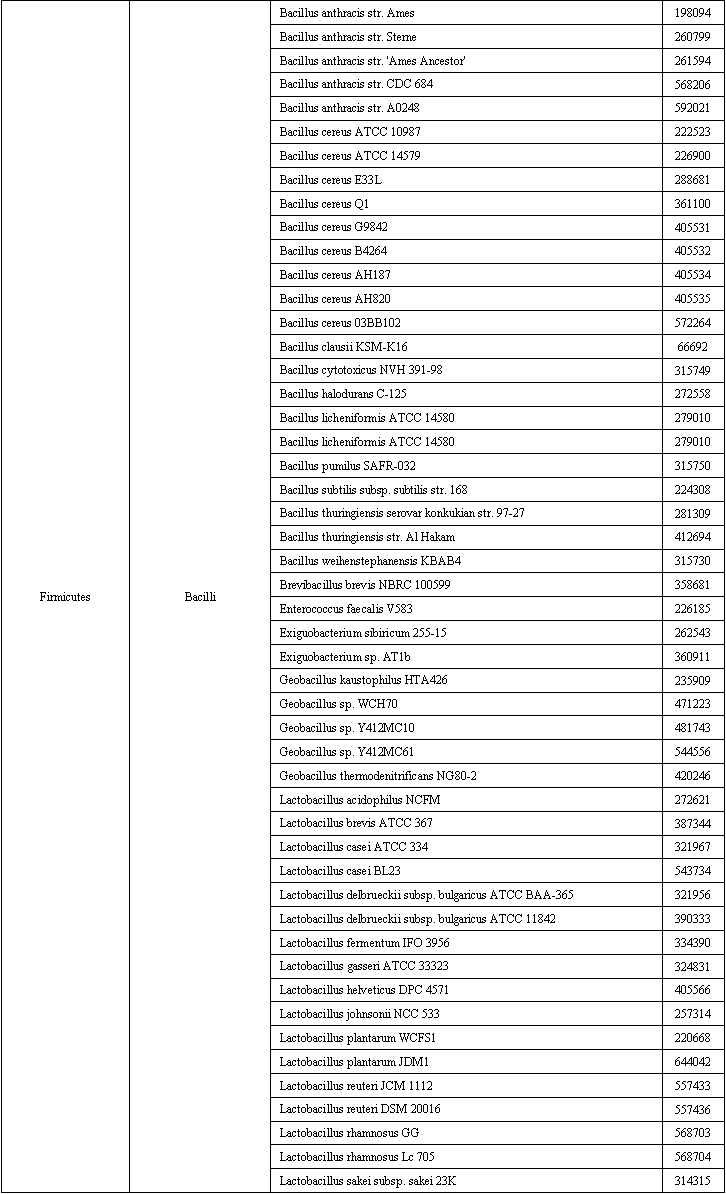


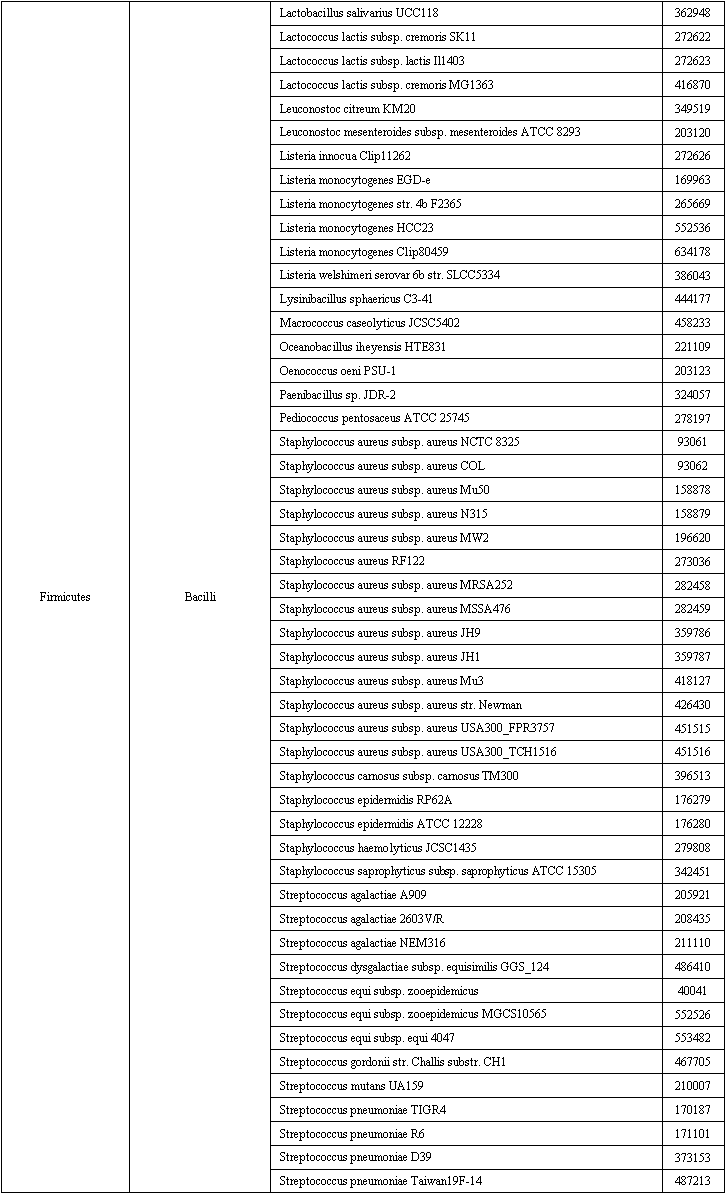


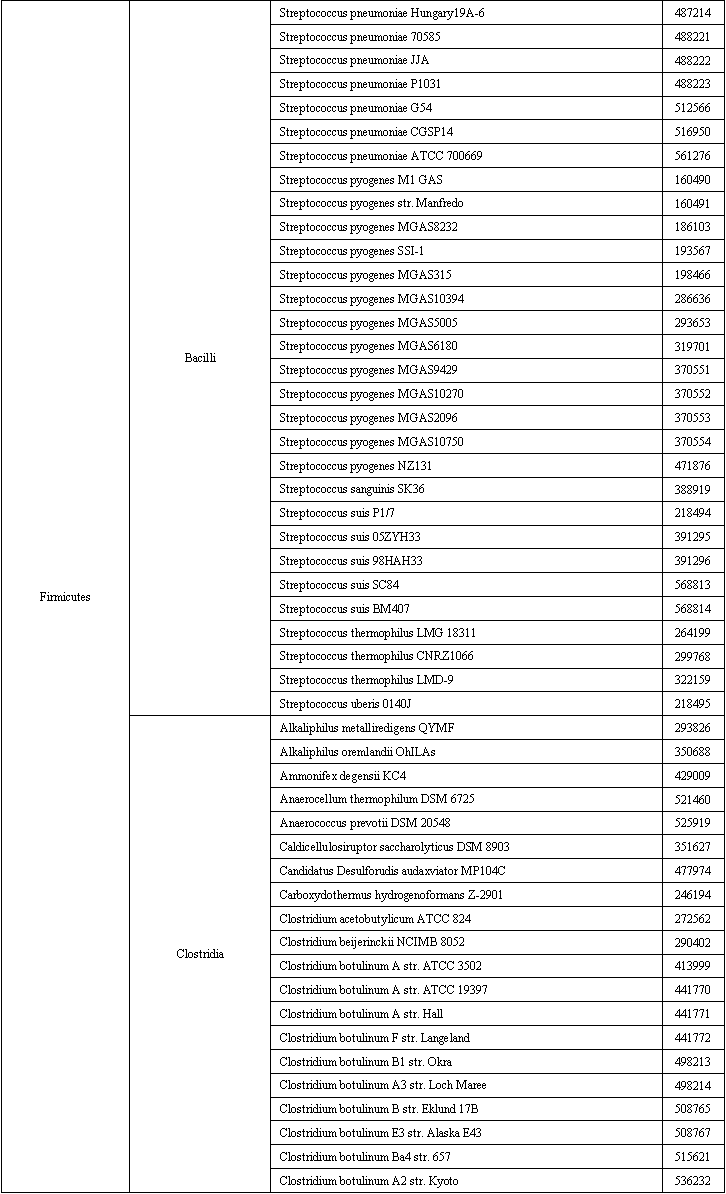


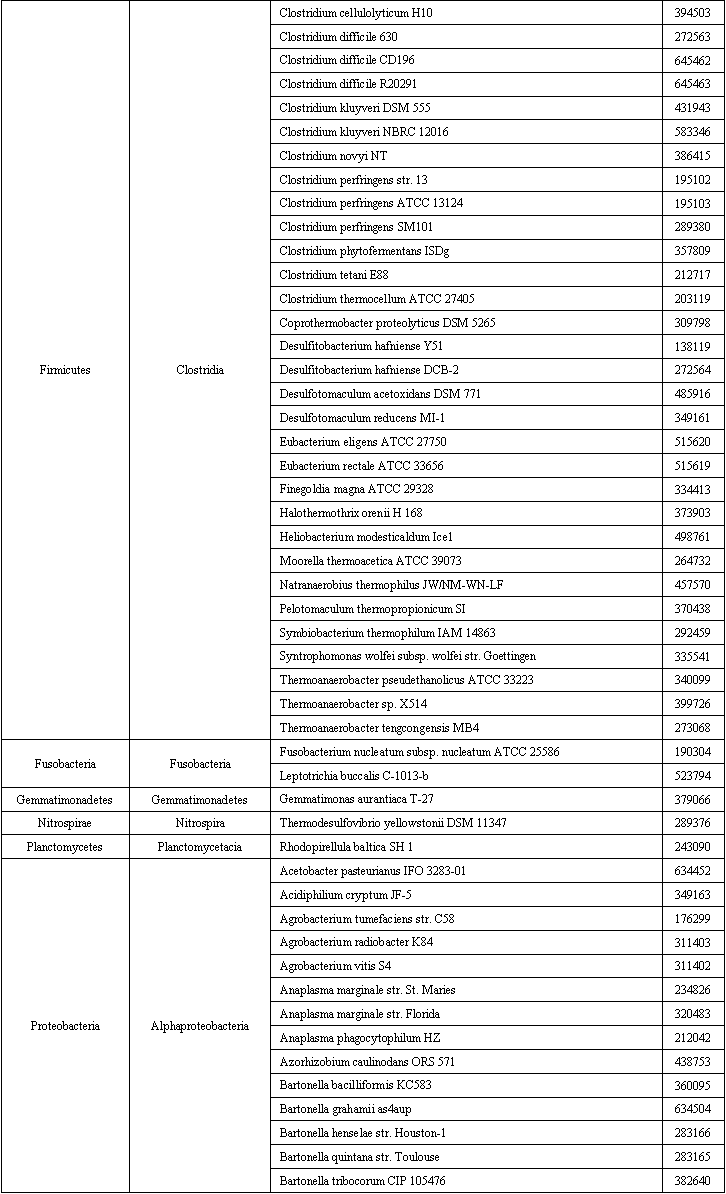


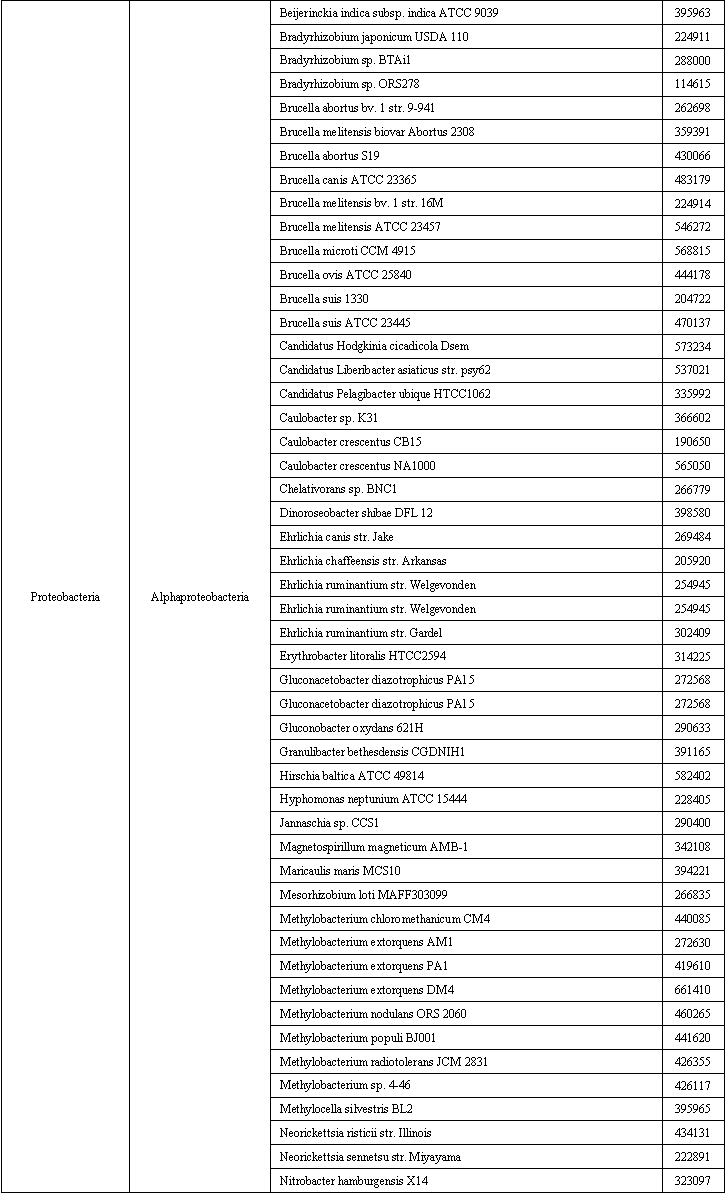


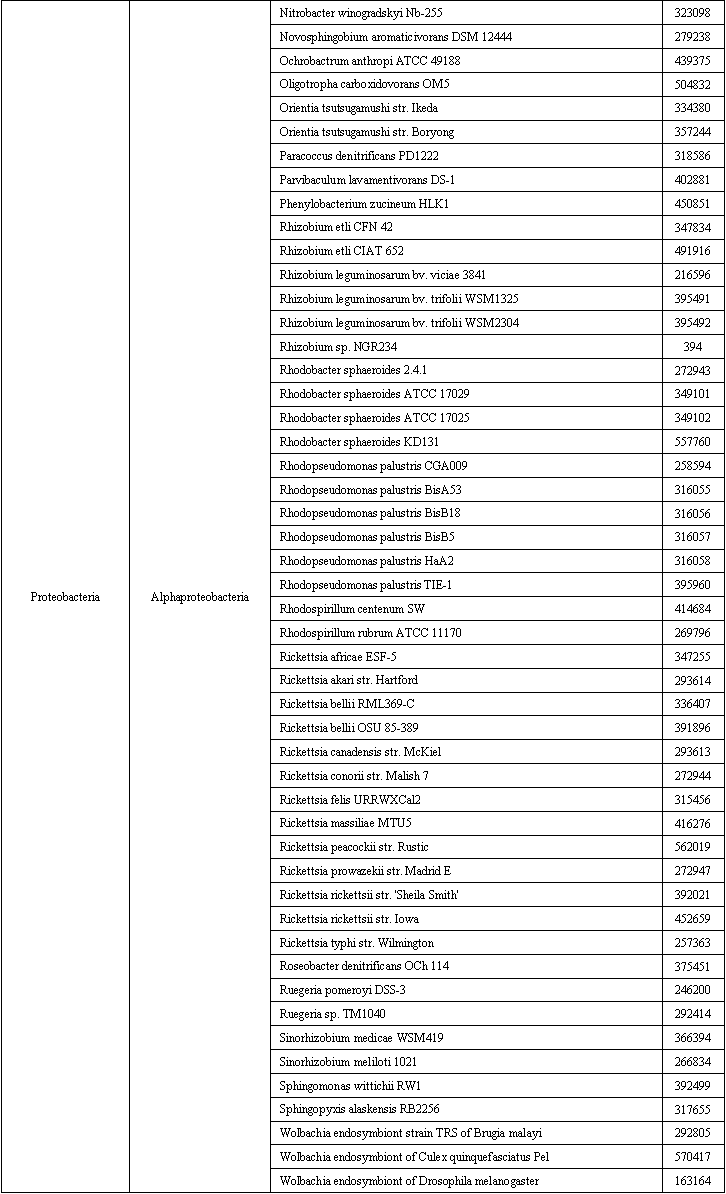


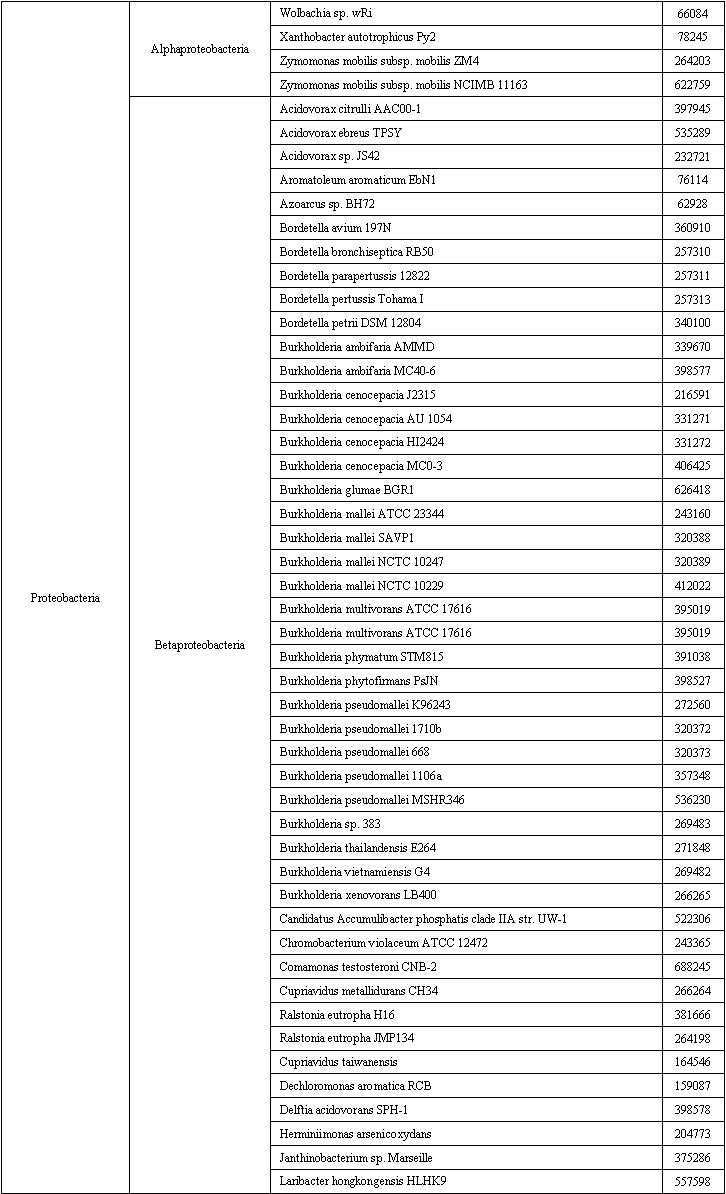


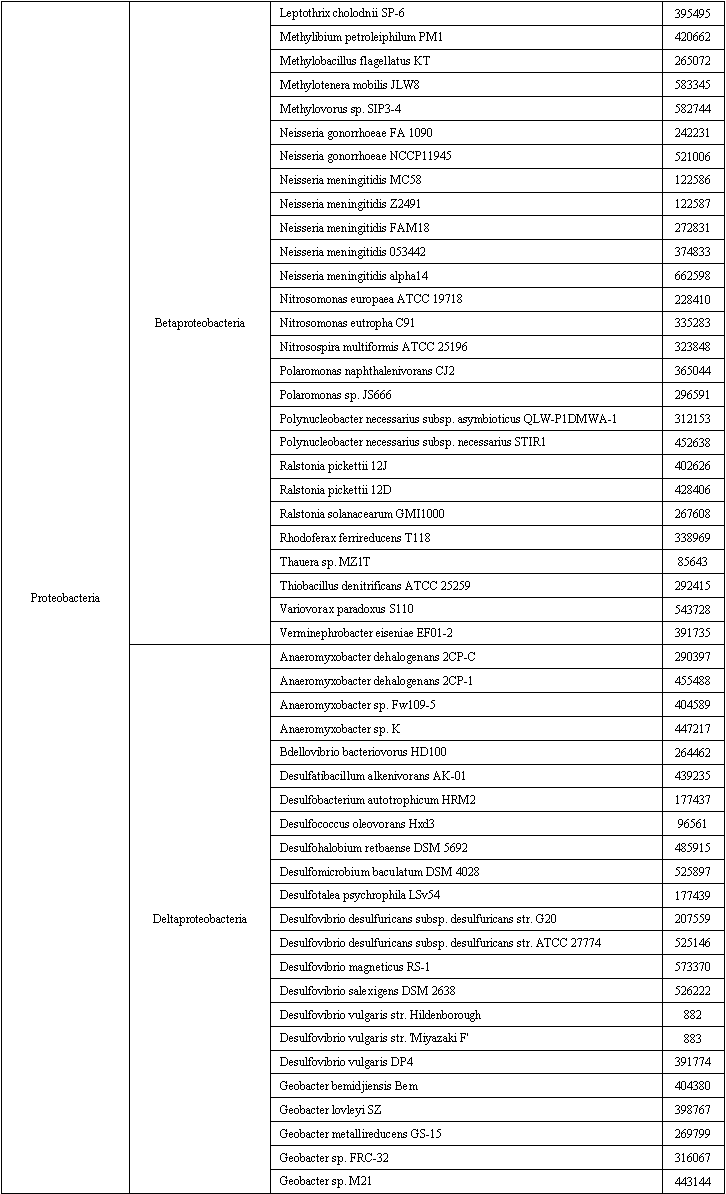


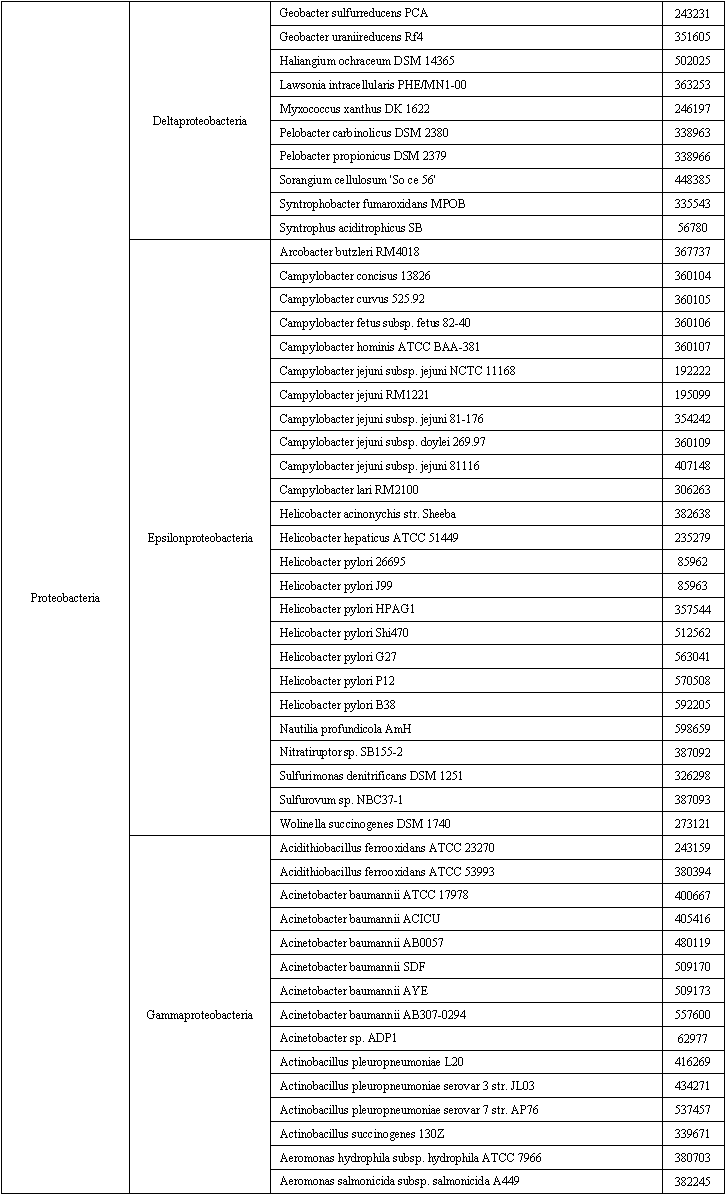


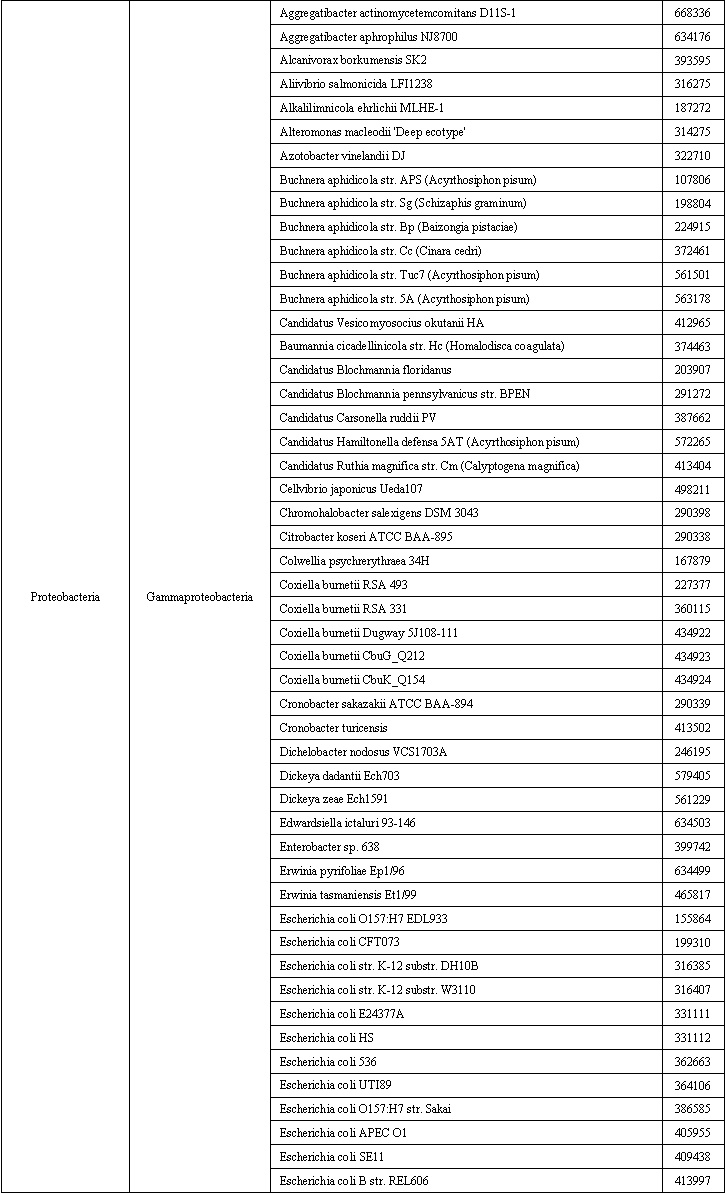


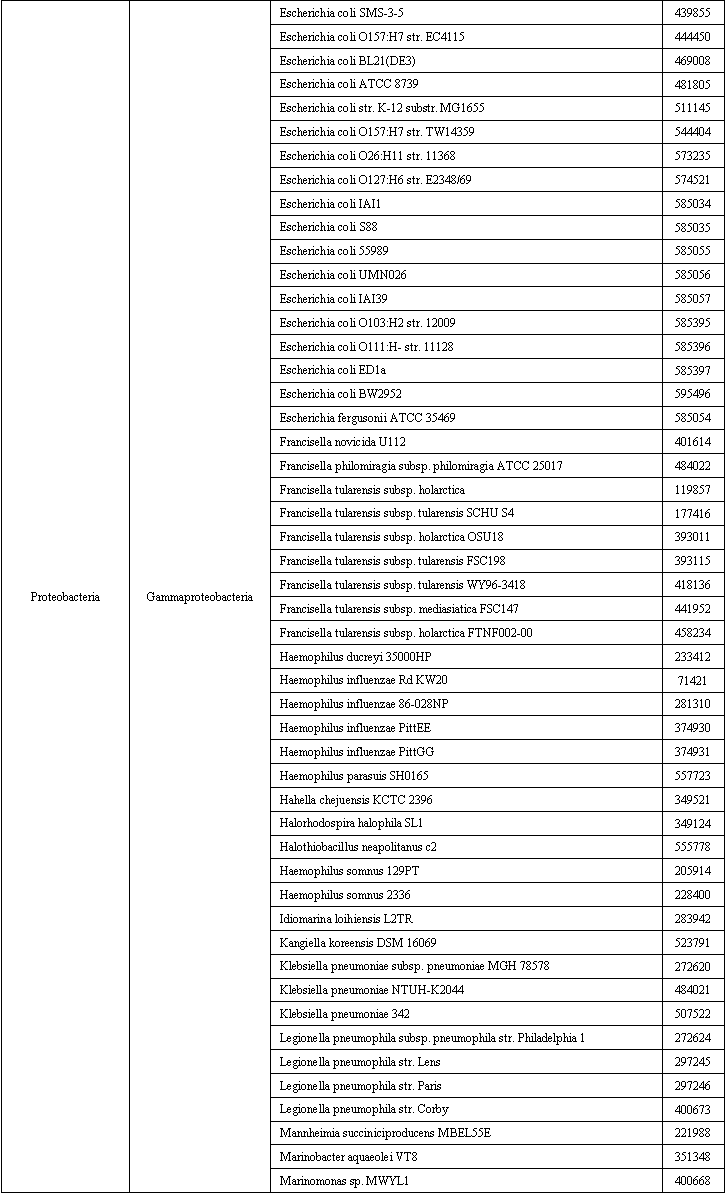


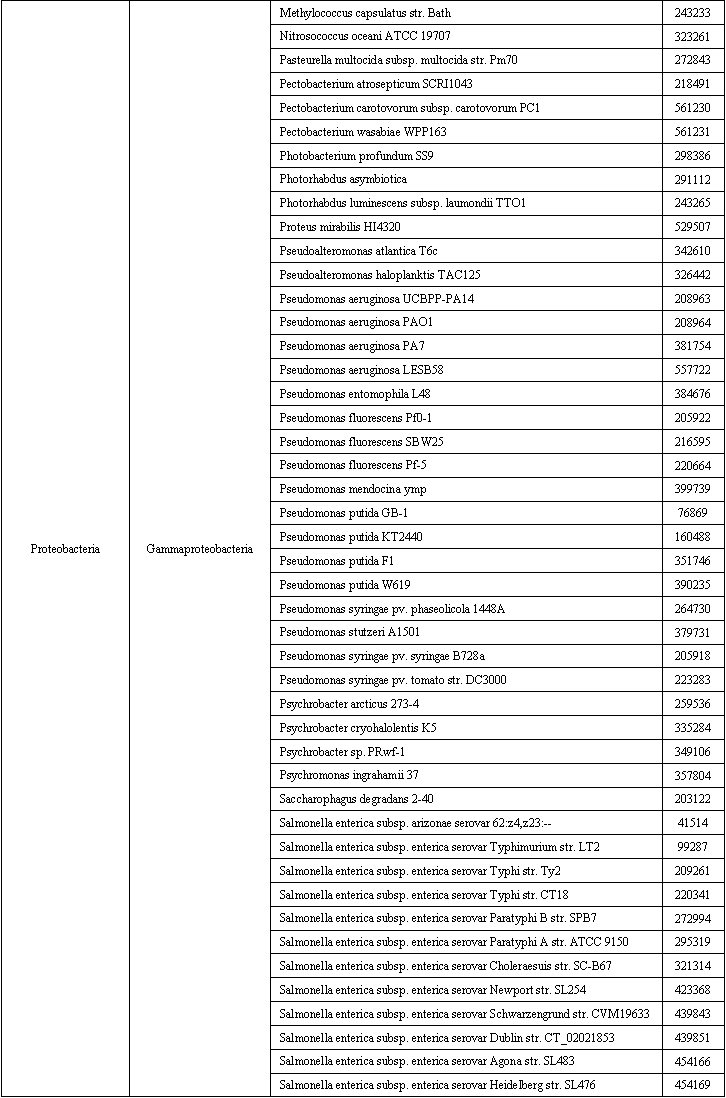


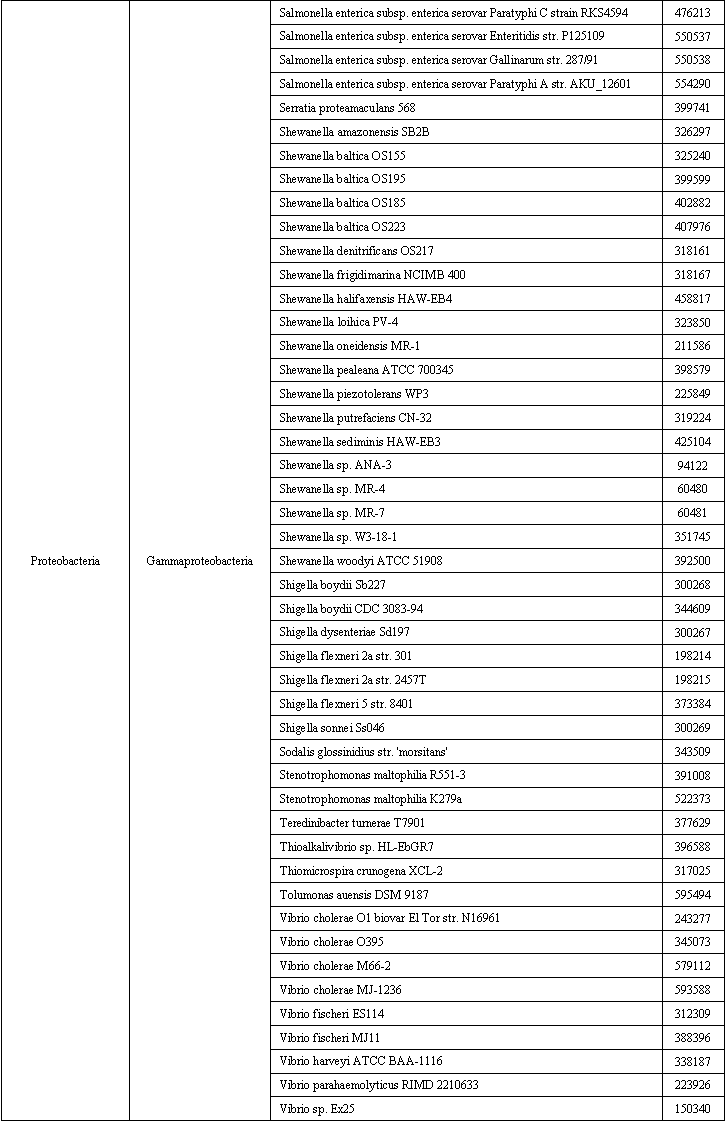


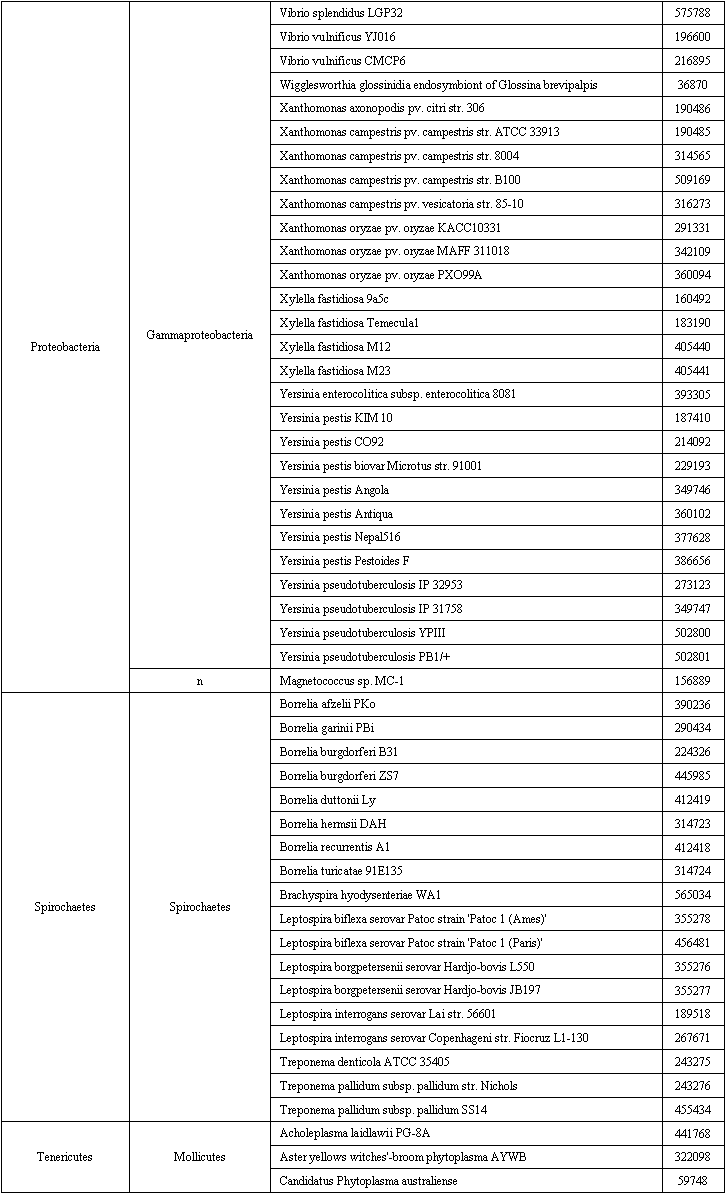


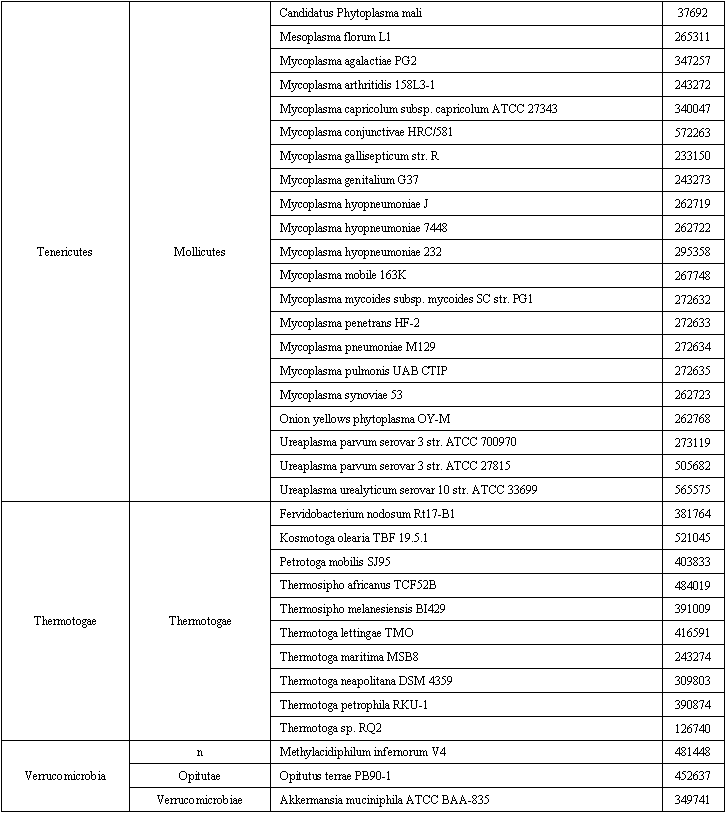

Supplement: Additional file 1 — Eubacterial organisms used in this detection. [file 1471-2148-11-356-S1.DOC]

**Additional file** **2** Archebacterial organisms used in this detection


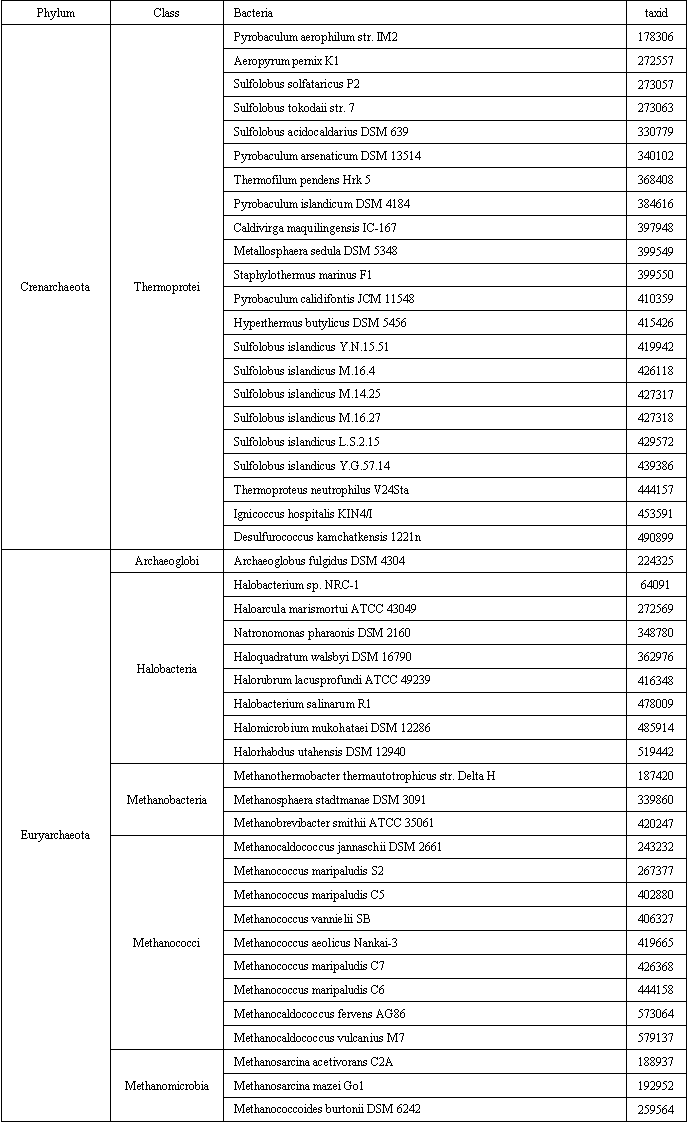


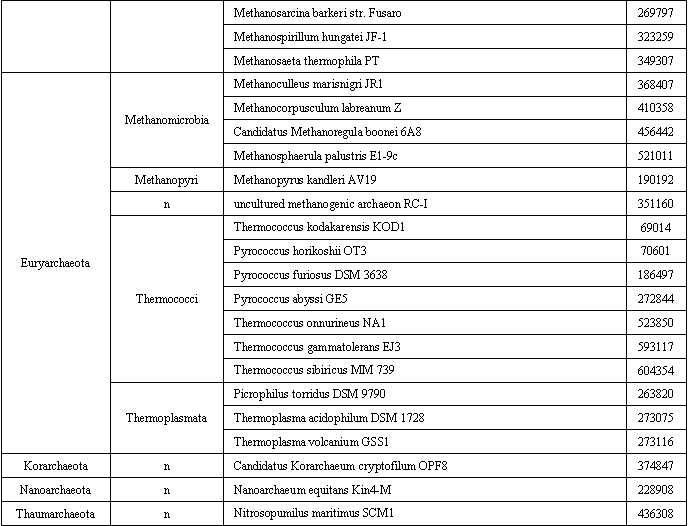

Supplement: Additional file 2 — Archebacterial organisms used in this detection. [file 1471-2148-11-356-S2.DOC]

**Additional file 3** Other organisms used in this detection.


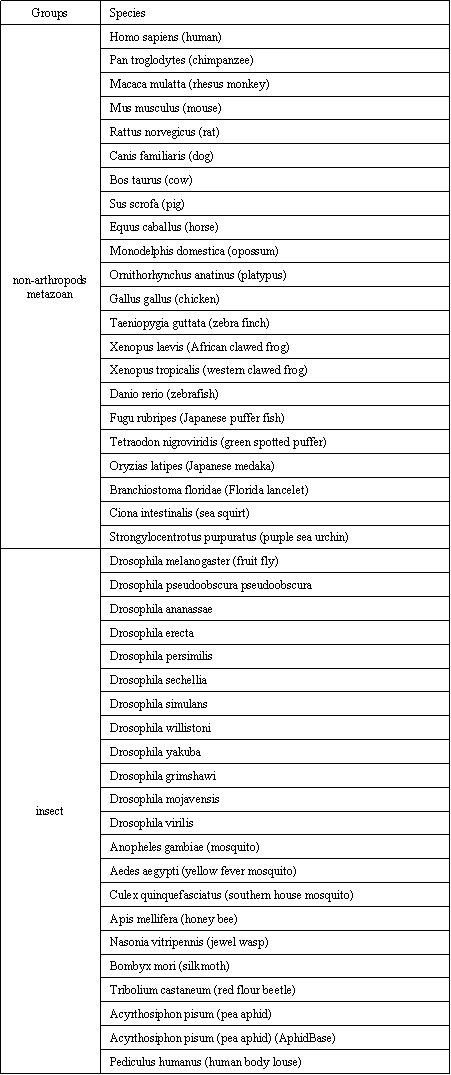


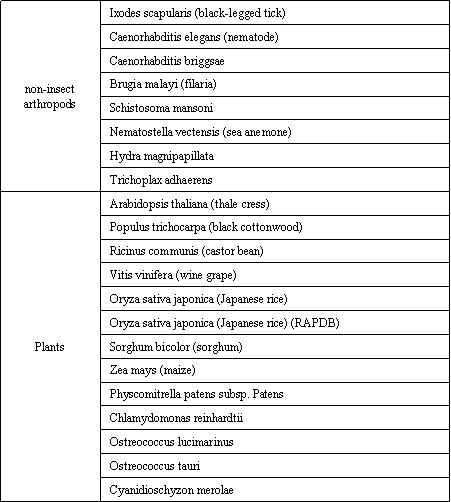


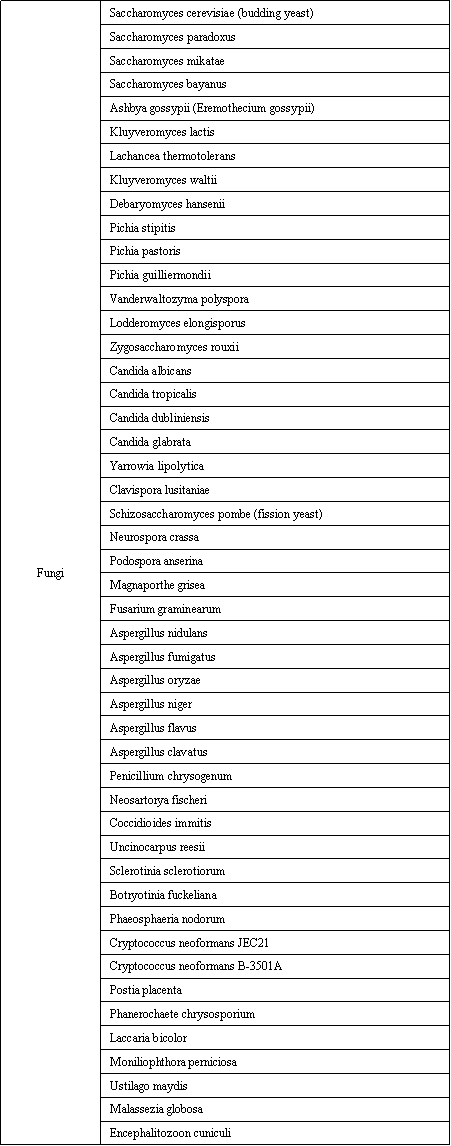


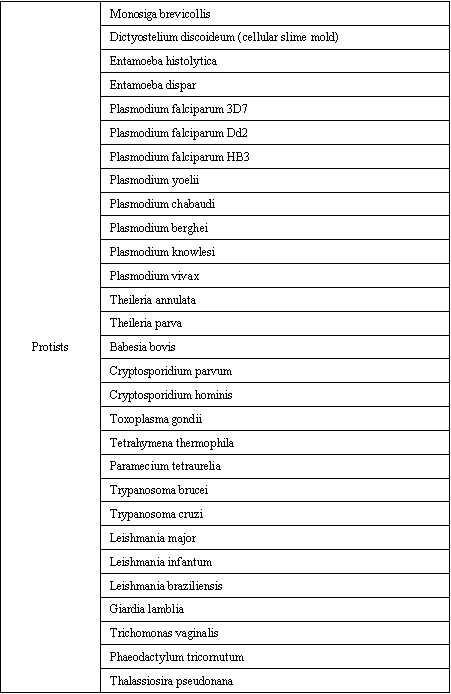

Supplement: Additional file 3 — Other organisms used in this detection. [file 1471-2148-11-356-S3.DOC]
