# Supplementary material for: Pathogen-origin horizontally transferred genes contribute to the evolution of Lepidopteran insects
Source: BMC Evol Biol. 2011 Dec 12;11:356. doi: 10.1186/1471-2148-11-356 (PMC3252269; doi:10.1186/1471-2148-11-356)
Supplement: Additional file 4 — Detected doubtful sequences in honeybee. [file 1471-2148-11-356-S4.DOC]

**Additional file** **4** Detected doubtful sequences in honeybee.

| Honeybee protein sequences | | Top blast hit in bacteria | | | |
| --- | --- | --- | --- | --- | --- |
| ID (NCBI) | ID (BeeBase) | E value | Identity | Species | ID (NCBI) |
| XP_001123274.1 | GB10027-PA | 5.00E-77 | 47.1 | Methylocella silvestris BL2 | YP_002363258 |
| XP_001123111.1 | GB10256-PA | 7.00E-83 | 70.0 | Lactobacillus helveticus DPC 4571 | YP_001577058 |
| XP_001123275.1 | GB10527-PA | 1.00E-152 | 71.2 | Acinetobacter sp ADP1 | YP_044878 |
| XP_001121058.1 | GB10962-PA | 1.00E-122 | 64.7 | Acetobacter pasteurianus IFO 3283 01 | YP_003186770 |
| XP_624275.1 | GB11094-PA | 1.00E-108 | 66.3 | Gluconacetobacter diazotrophicus PAl 5 FAPERJ | YP_001601561 |
| XP_001122890.1 | GB11109-PA | 1.00E-132 | 69.1 | Neisseria meningitidis MC58 | NP_274579 |
| XP_001123229.1 | GB11185-PA | 1.00E-146 | 67.0 | Clostridium beijerinckii NCIMB 8052 | YP_001311719 |
| XP_001123352.1 | GB11841-PA | 1.00E-114 | 73.7 | Acinetobacter baumannii AB0057 | YP_002319876 |
| XP_001122852.1 | GB11888-PA | 3.00E-64 | 59.3 | Lactobacillus helveticus DPC 4571 | YP_001576603 |
| XP_001122442.1 | GB12141-PA | 0 | 80.3 | Neisseria gonorrhoeae FA 1090 | YP_208302 |
| XP_001119935.1 | GB12333-PA | 2.00E-67 | 58.5 | Neisseria meningitidis 053442 | YP_001598541 |
| XP_001123024.1 | GB12376-PA | 2.00E-62 | 49.6 | Granulobacter bethesdensis CGDNIH1 | YP_744579 |
| XP_624954.1 | GB12615-PA | 1.00E-133 | 70.9 | Xanthomonas campestris vesicatoria 85-10 | YP_362765 |
| XP_001122950.1 | GB13288-PA | 1.00E-106 | 59.5 | Haemophilus somnus 129PT | YP_719353 |
| XP_001122737.1 | GB13584-PA | 1.00E-111 | 47.3 | Pseudomonas putida W619 | YP_001751481 |
| XP_001123159.1 | GB14131-PA | 1.00E-105 | 66.5 | Gluconobacter oxydans 621H | YP_192466 |
| XP_001122636.1 | GB14322-PA | 1.00E-112 | 68.6 | Pediococcus pentosaceus ATCC 25745 | YP_804172 |
| XP_001123208.1 | GB14449-PA | 1.00E-118 | 70.6 | Neisseria meningitidis MC58 | NP_274979 |
| XP_001123075.1 | GB14661-PA | 5.00E-90 | 73.6 | Neisseria meningitidis 053442 | YP_001598485 |
| XP_001122911.1 | GB14662-PA | 3.00E-76 | 67.2 | Citrobacter koseri ATCC BAA-895 | YP_001454869 |
| XP_001121973.1 | GB14716-PA | 4.00E-86 | 57.6 | Lactobacillus brevis ATCC 367 | YP_794427 |
| XP_001123061.1 | GB14728-PA | 1.00E-156 | 66.5 | Neisseria meningitidis FAM18 | YP_974913 |
| XP_001123034.1 | GB14768-PA | 2.00E-88 | 61.8 | Acetobacter pasteurianus IFO 3283 01 | YP_003188278 |
| XP_001123090.1 | GB14917-PA | 4.00E-56 | 63.9 | Pectobacterium carotovorum PC1 | YP_003015975 |
| XP_624542.1 | GB15756-PA | 2.00E-95 | 75.7 | Neisseria meningitidis alpha14 | YP_003083878 |
| XP_001122282.1 | GB15788-PA | 2.00E-87 | 80.6 | Marinobacter aquaeolei VT8 | YP_961094 |
| XP_001123341.1 | GB15852-PA | 6.00E-72 | 61.0 | Methylobacillus flagellatus KT | YP_546198 |
| XP_001123147.1 | GB16347-PA | 1.00E-75 | 55.0 | Gluconacetobacter diazotrophicus PAl 5 FAPERJ | YP_001602120 |
| XP_001120646.1 | GB16374-PA | 8.00E-51 | 62.5 | Ralstonia solanacearum | NP_518681 |
| XP_001120932.1 | GB16797-PA | 8.00E-62 | 52.4 | Pediococcus pentosaceus ATCC 25745 | YP_804111 |
| XP_001123121.1 | GB17115-PA | 5.00E-57 | 56.8 | Acinetobacter baumannii ACICU | YP_001848109 |
| XP_001123078.1 | GB17350-PA | 1.00E-108 | 60.9 | Neisseria meningitidis Z2491 | YP_002341801 |
| XP_001122889.1 | GB17392-PA | 2.00E-88 | 71.5 | Lactobacillus casei | YP_001988574 |
| XP_001123273.1 | GB18066-PA | 1.00E-132 | 79.3 | Acinetobacter sp ADP1 | YP_044880 |
| XP_625064.1 | GB18103-PA | 0 | 81.1 | Acetobacter pasteurianus IFO 3283 01 | YP_003187165 |
| XP_001122764.1 | GB18134-PA | 1.00E-61 | 71.1 | Ralstonia metallidurans CH34 | YP_584356 |
| XP_001122770.1 | GB18701-PA | 1.00E-113 | 86.0 | Neisseria meningitidis alpha14 | YP_003083445 |
| XP_001122129.1 | GB18829-PA | 4.00E-68 | 83.9 | Acetobacter pasteurianus IFO 3283 01 | YP_003186751 |
| XP_001123251.1 | GB19045-PA | 1.00E-42 | 56.4 | Tolumonas auensis DSM 9187 | YP_002892486 |
| XP_001122941.1 | GB19339-PA | 1.00E-76 | 63.7 | Acetobacter pasteurianus IFO 3283 01 | YP_003188252 |
| XP_001123346.1 | GB19893-PA | 9.00E-41 | 64.3 | Gluconacetobacter diazotrophicus PAl 5 FAPERJ | YP_001602461 |
| XP_001122528.1 | GB19926-PA | 1.00E-164 | 80.7 | Lactobacillus acidophilus NCFM | YP_193739 |
| XP_001121855.1 | GB20124-PA | 1.00E-109 | 64.1 | Yersinia enterocolitica 8081 | YP_001004691 |
| XP_001122460.1 | GB20143-PA | 5.00E-86 | 55.0 | Gluconacetobacter diazotrophicus PAl 5 JGI | YP_002275018 |
| XP_001119873.1 | no | 1.00E-103 | 68.7 | Serratia proteamaculans 568 | YP_001478808 |
| XP_001119933.1 | no | 7.00E-67 | 59.6 | Enterobacter 638 | YP_001178361 |
| XP_001120185.1 | no | 1.00E-53 | 96.1 | Neisseria gonorrhoeae FA 1090 | YP_208874 |
| XP_001120301.1 | no | 2.00E-64 | 70.7 | Proteus mirabilis | YP_002153048 |
| XP_001121294.1 | no | 6.00E-58 | 72.0 | Chlorobium phaeobacteroides BS1 | YP_001960462 |
| XP_001121469.1 | no | 1.00E-131 | 69.3 | Klebsiella pneumoniae MGH 78578 | YP_001334438 |
| XP_001121854.1 | no | 1.00E-141 | 70.7 | Laribacter hongkongensis HLHK9 | YP_002795115 |
| XP_001121878.1 | no | 5.00E-93 | 86.7 | Haemophilus influenzae | NP_438694 |
| XP_001122303.1 | no | 2.00E-54 | 50.0 | Burkholderia xenovorans LB400 | YP_552673 |
| XP_001122410.1 | no | 5.00E-98 | 54.5 | Slackia heliotrinireducens DSM 20476 | YP_003143413 |
| XP_001122490.1 | no | 1.00E-105 | 62.1 | Pediococcus pentosaceus ATCC 25745 | YP_804781 |
| XP_001122589.1 | no | 8.00E-74 | 72.9 | Magnetospirillum magneticum AMB-1 | YP_421186 |
| XP_001122601.1 | no | 1.00E-163 | 77.4 | Gluconacetobacter diazotrophicus PAl 5 FAPERJ | YP_001601078 |
| XP_001122677.1 | no | 1.00E-102 | 82.9 | Enterobacter 638 | YP_001178361 |
| XP_001122731.1 | no | 1.00E-159 | 56.9 | Gluconacetobacter diazotrophicus PAl 5 FAPERJ | YP_001602274 |
| XP_001122758.1 | no | 3.00E-79 | 70.2 | Neisseria meningitidis alpha14 | YP_003082685 |
| XP_001122797.1 | no | 4.00E-44 | 78.4 | Neisseria meningitidis FAM18 | YP_974515 |
| XP_001122828.1 | no | 6.00E-54 | 51.7 | Pelobacter carbinolicus | YP_358241 |
| XP_001122834.1 | no | 5.00E-85 | 72.2 | Teredinibacter turnerae T7901 | YP_003073984 |
| XP_001122859.1 | no | 1.00E-146 | 80.1 | Lactobacillus helveticus DPC 4571 | YP_001577239 |
| XP_001122892.1 | no | 3.00E-54 | 82.4 | Lactobacillus gasseri ATCC 33323 | YP_814154 |
| XP_001122924.1 | no | 4.00E-59 | 94.1 | Lactobacillus helveticus DPC 4571 | YP_001577168 |
| XP_001122990.1 | no | 0 | 72.3 | Neisseria meningitidis Z2491 | YP_002341941 |
| XP_001123014.1 | no | 1.00E-115 | 73.9 | Serratia proteamaculans 568 | YP_001479820 |
| XP_001123026.1 | no | 1.00E-117 | 71.2 | Neisseria meningitidis Z2491 | YP_002341941 |
| XP_001123103.1 | no | 1.00E-60 | 85.7 | Acetobacter pasteurianus IFO 3283 01 | YP_003188159 |
| XP_001123171.1 | no | 1.00E-160 | 90.4 | Bordetella avium 197N | YP_787230 |
| XP_001123225.1 | no | 1.00E-86 | 67.9 | Neisseria gonorrhoeae FA 1090 | YP_207570 |
| XP_001123311.1 | no | 4.00E-84 | 78.9 | Neisseria meningitidis MC58 | NP_274209 |
| XP_001123315.1 | no | 0 | 85.3 | Lactobacillus delbrueckii bulgaricus | YP_619095 |
| XP_001123350.1 | no | 1.00E-165 | 84.0 | Gluconacetobacter diazotrophicus PAl 5 FAPERJ | YP_001602356 |
| XP_001123360.1 | no | 2.00E-53 | 67.1 | Klebsiella pneumoniae 342 | YP_002237243 |
| XP_624992.2 | no | 1.00E-159 | 72.6 | Lactobacillus plantarum | NP_786060 |
| XP_001121202.1 | no | 1.00E-148 | 81.8 | Dickeya dadantii Ech703 | YP_002986738 |
| XP_001121393.1 | no | 1.00E-123 | 66.1 | Lactobacillus plantarum JDM1 | YP_003064442 |
