# Supplementary material for: Pathogen-origin horizontally transferred genes contribute to the evolution of Lepidopteran insects
Source: BMC Evol Biol. 2011 Dec 12;11:356. doi: 10.1186/1471-2148-11-356 (PMC3252269; doi:10.1186/1471-2148-11-356)
Supplement: Additional file 5 — Homologous sequences of silkworm HTGs in other Lepidopteran insects. [file 1471-2148-11-356-S5.PDF]

Additional file 5 Homologous sequences of silkworm HTGs in other Lepidopteran insects

Gene ID	Bombycoidea		Papilionoidea		Noctuoidea		Pyraloidea		Tortricoidea
	Saturniidae	Sphingidae	Nymphalidae	Papilionidae	Noctuidae	Arctiidae	Crambidae	Pyralidae	Tortricidae
BGIBMGA000070			*Bicyclus anynana*<br>(GE656914)		*Striacosta albicosta*<br>(EZ583711) (EZ588036)	*Hyphantria cunea*<br>(AF497847)<br>(AF497848)		*Plodia*<br>*interpunctella*<br>(AF092741)<br>*Galleria mellonella*<br>(U22425)	
BGIBMGA001284	*Antheraea mylitta*<br>(AMC00860_1)				*Striacosta albicosta*<br>(EZ584277) (EZ586683)<br>*Helicoverpa armigera* (DQ875236,<br>DQ875249)<br>*Spodoptera frugiperda* (SFC03690_2)				*Choristoneura*<br>*fumiferana* (FE271381)
BGIBMGA002521			*Bicyclus anynana*<br>(GE663569)		*Trichoplusia ni* (CF259326)<br>*Heliothis virescens* (HO053951)<br>*Striacosta albicosta* (FP372718; FP360470)				
BGIBMGA005555<br>BGIBMGA005696		*Manduca sexta*<br>(GQ293364)<br>(GQ293363)	*Heliconius erato emma*<br>(GU582903)<br>*Heliconius melpomene*<br>*rosina* (GU077782)<br>*Bicyclus anynana*<br>(GE662769, GE667386)		*Striacosta albicosta* (EZ588214)<br>*Helicoverpa armigera* (EF600050)<br>*Heliothis virescens*<br>(GR971067)(GT194715)<br>(GT194716)(GR971931)(GT055946)<br>*Trichoplusia ni* (FF379051; FF379471)		*Ostrinia nubilalis*<br>(GH993988)(GH991355)	*Plodia*<br>*interpunctella*<br>(EB824649)	*Epiphyas postvittana*<br>(EV811092,<br>EV806210)
BGIBMGA005615	*Antheraea mylitta*<br>(AMC01280_1, AMC02116_1)<br>*Samia cynthia ricini*<br>(DC860239)(DC868144)<br>*Antheraea assama* (FG226624,<br>FG220658)		*Heliconius numata*<br>(FP885880)<br>*Bicyclus anynana*<br>(BAC03175_1)<br>*Danaus plexippus*<br>(EY271965)		*Striacosta albicosta*<br>(EZ582017) (EZ583889)<br>*Helicoverpa armigera* (HAC00282_1)<br>*Spodoptera frugiperda*<br>(SFC00263_2, SFC10591_1)<br>*Heliothis virescens* (GT055200)<br>*Trichoplusia ni* (FF368874)		*Ostrinia nubilalis*<br>(GH994317)(GH996649)		*Choristoneura*<br>*fumiferana*<br>(FC974344)<br>*Epiphyas postvittana*<br>(EV807109)
BGIBMGA007146		*Manduca sexta*<br>(GR922050)	*Bicyclus anynana*<br>(GE659866)		*Striacosta albicosta* (EZ585074, EZ585412,<br>EZ581764, EZ585746)<br>*Heliothis virescens* (GT055197)				*Choristoneura*<br>*fumiferana*<br>(FC972431)
BGIBMGA007766<br>BGIBMGA007767	*Samia cynthia ricini* (DC861465)	*Manduca sexta*<br>(MSC01302_1)	*Bicyclus anynana*<br>(GE672459, GE654201)<br>(GE654202)		*Striacosta albicosta* (EZ583353)<br>*Spodoptera frugiperda* (SFC02785_1)<br>*Heliothis virescens* (GT055366)<br>*Trichoplusia ni* (FF369839)		*Ostrinia nubilalis*<br>(GH997503)(GH988444)		*Choristoneura*<br>*fumiferana* (FE273873)
BGIBMGA008215	*Antheraea assama* (FG211456)	*Manduca sexta*<br>(GR920753,GR920756)							
BGIBMGA008709	*Antheraea pernyi* (AB201279)<br>*Samia cynthia* (AB201280)<br>*Antheraea assama* (FE963475,<br>FE953746, FE960589)	*Manduca sexta*<br>(DQ288140)<br>*Agrius convolvuli*<br>(AB201282)	*Heliconius melpomene*<br>(HMC00756_1,<br>HMC02616_1)<br>*Heliconius erato*<br>(HEC03581_1)<br>*Bicyclus anynana*<br>(BAC01614_1)<br>*Heliconius melpomene*<br>(CX700792, ES586194,<br>ES585647)	*Papilio*<br>*dardanus*<br>(PDC00118_1)<br>*Papilio xuthus*<br>(DK968701)	*Striacosta albicosta* (EZ590656, EZ59484)<br>*Spodoptera littoralis* (DQ185388)<br>*Spodoptera exempta* (DQ185398)<br>*Spodoptera exigua* (GU371869)<br>*Mamestra brassicae* (DQ185397)<br>*Mythimna separata* (AB201283)<br>*Spodoptera frugiperda*<br>(FP357414, FP357258)<br>*Heliothis virescens* (GT209928, GT187728,<br>GT187729)			*Ostrinia furnacalis*<br>(AB201281)	*Choristoneura*<br>*fumiferana* (FE273578,<br>FE272008, FE272037,<br>FE273151)
BGIBMGA010285<br>BGIBMGA010866	*Samia cynthia ricini* (DC870719,<br>DC866254)(DC870705)<br>*Antheraea assama* (FG208221)				*Striacosta albicosta* (EZ581487)<br>*Spodoptera frugiperda* (FP363125,<br>FP372574)<br>(FP367938)(FP364008)(FP360049)				
BGIBMGA011199<br>BGIBMGA011200<br>BGIBMGA011201<br>BGIBMGA011202<br>BGIBMGA011203<br>BGIBMGA011204		*Manduca sexta*<br>(GR922847)	*Heliconius melpomene*<br>(HMC00565_1)<br>*Danaus plexippus*<br>(EY263418)(EY264747)		*Striacosta albicosta* (EZ591942)<br>*Spodoptera frugiperda* (SFC01682_1)<br>*Trichoplusia ni* (FF368873)<br>*Heliothis virescens*<br>(GT134130)(GR970402)				*Choristoneura*<br>*fumiferana* (FE271949)<br>*Epiphyas postvittana*<br>(EV806761,<br>EV807414)
BGIBMGA012123	*Samia cynthia ricini*<br>(DC868421)	*Manduca sexta*<br>(MSC00474_1)	*Heliconius erato*<br>(HEC09288_1)<br>*Danaus plexippus*<br>(EY270341)(EY270777)<br>(EY269713)(EY267551)<br>*Bicyclus anynana*<br>(GE678403, GE678404)<br>(GE682125, GE682124)		*Striacosta albicosta* EZ590849)<br>*Heliothis virescens*<br>(EY121932)(GR965887)<br>*Spodoptera frugiperda*<br>(FP354875, FP359108)<br>(FP365714)(FP351655)<br>*Trichoplusia ni* (FF378281)				
BGIBMGA013995	*Antheraea assama* (FG205725)								
