# Supplementary material for: Pathogen-origin horizontally transferred genes contribute to the evolution of Lepidopteran insects
Source: BMC Evol Biol. 2011 Dec 12;11:356. doi: 10.1186/1471-2148-11-356 (PMC3252269; doi:10.1186/1471-2148-11-356)
Supplement: Additional file 6 — Multiple alignment of amino acid sequences and phylogenetic trees of detected HTGs. [file 1471-2148-11-356-S6.PDF]

Figure S1(a)

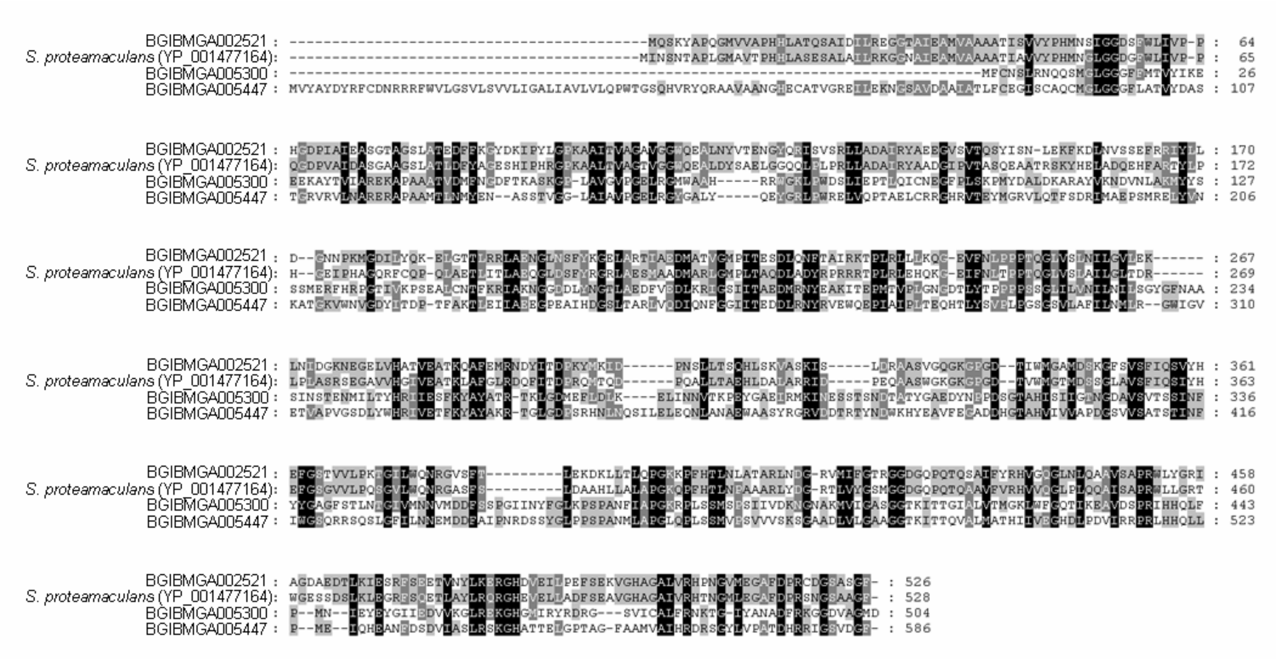

Figure S1(b)

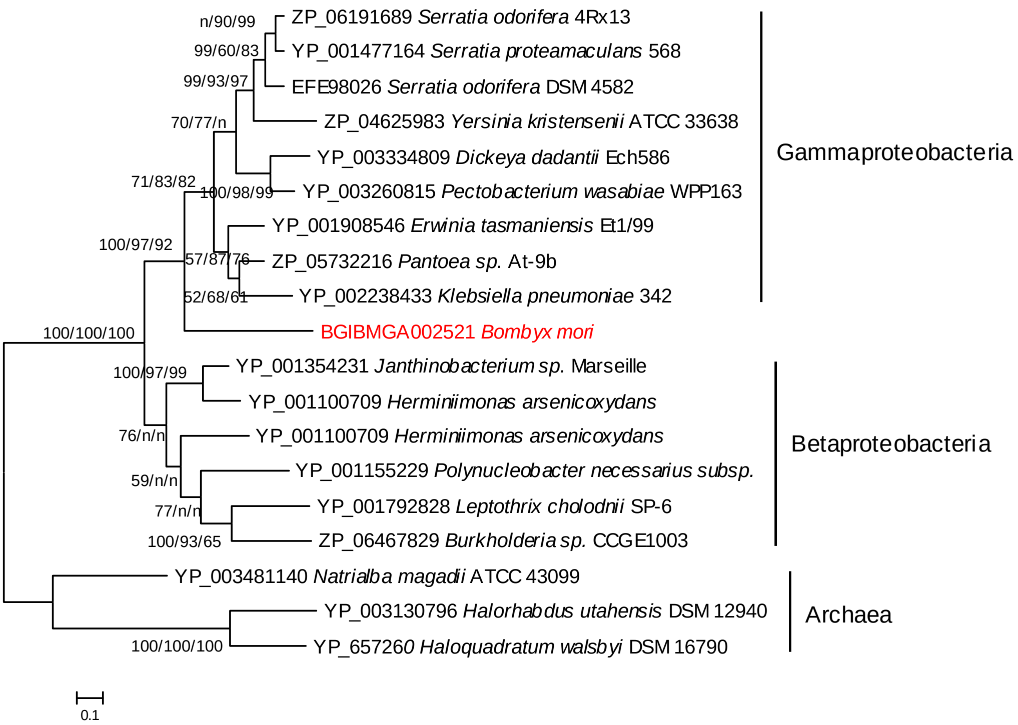

Figure S1 (a) Multiple alignment of amino acid sequences of BGIBMGA002521 and its homologs.

(b) Phylogenetic tree of BGIBMGA002521 and its homologs. Numbers beside nodes indicate supporting values in methods of BI/ML/NJ.

Figure S2(a)

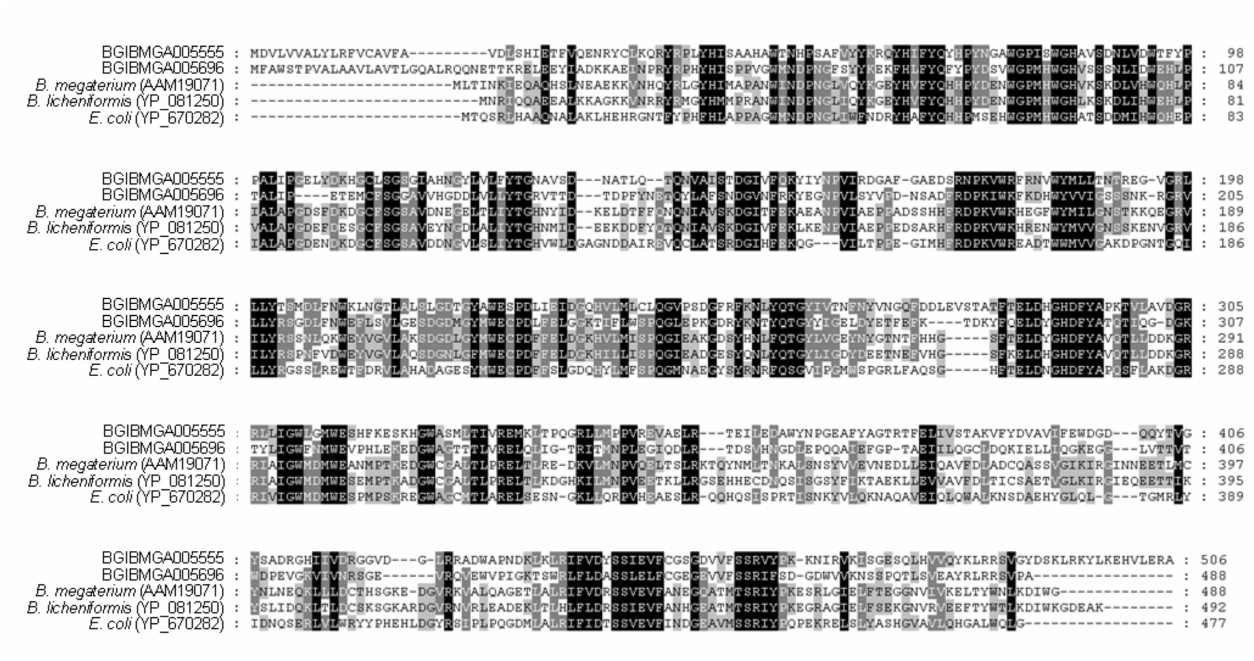

Figure S2(b)

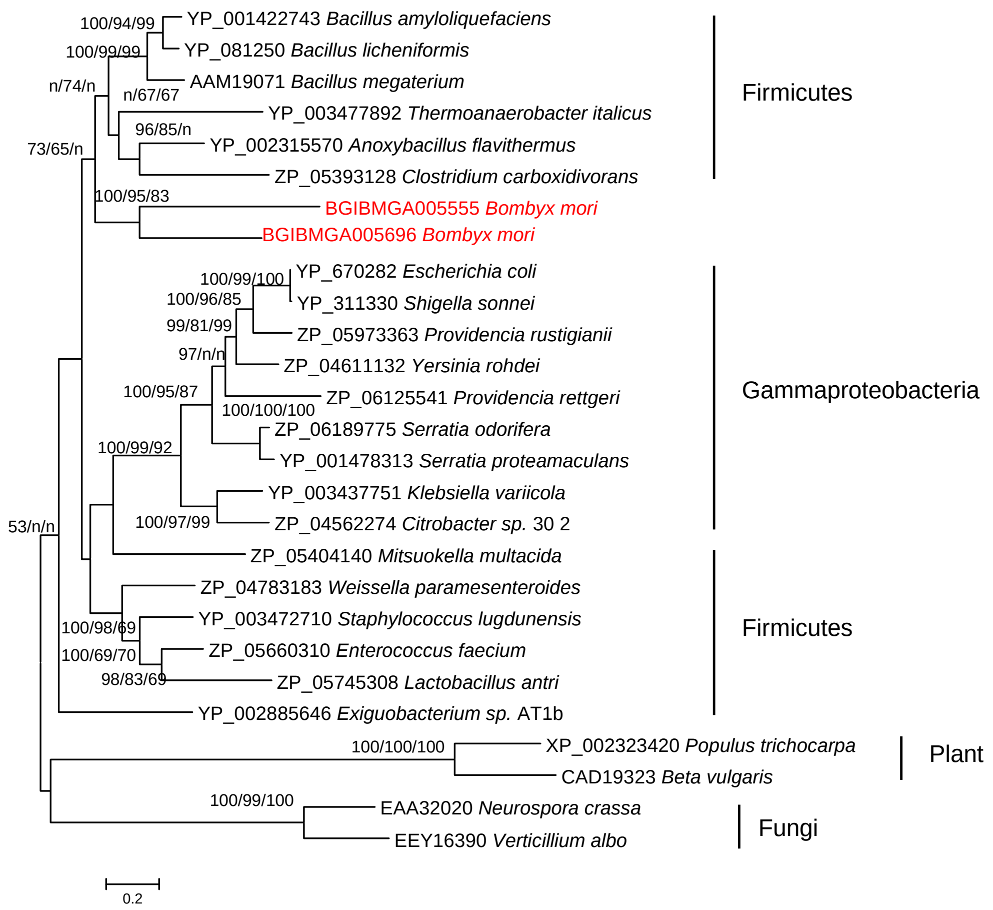

Figure S2 (a) Multiple alignment of amino acid sequences of BGIBMGA005555, BGIBMGA005696 and their homologs. (b) Phylogenetic tree of BGIBMGA005555, BGIBMGA005696 and their homologs. Numbers beside nodes indicate supporting values in methods of BI/ML/NJ.

Figure S3(a)

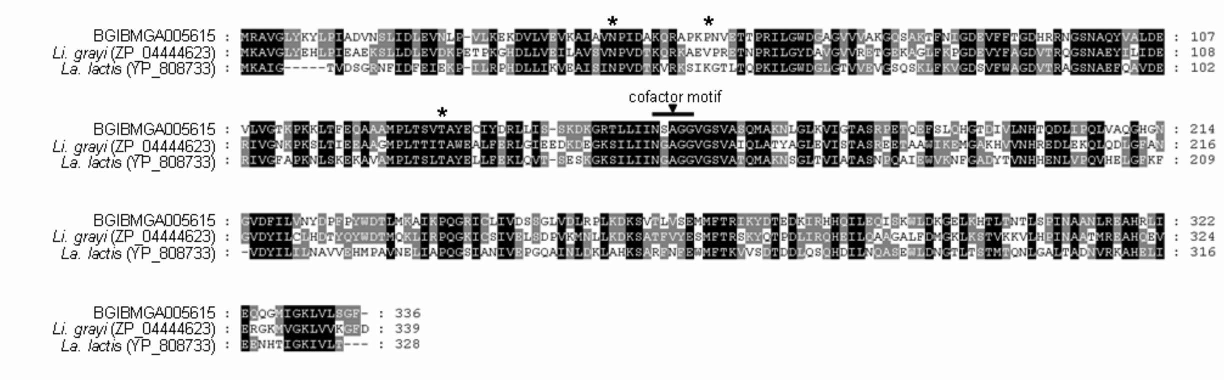

Figure S3(b)

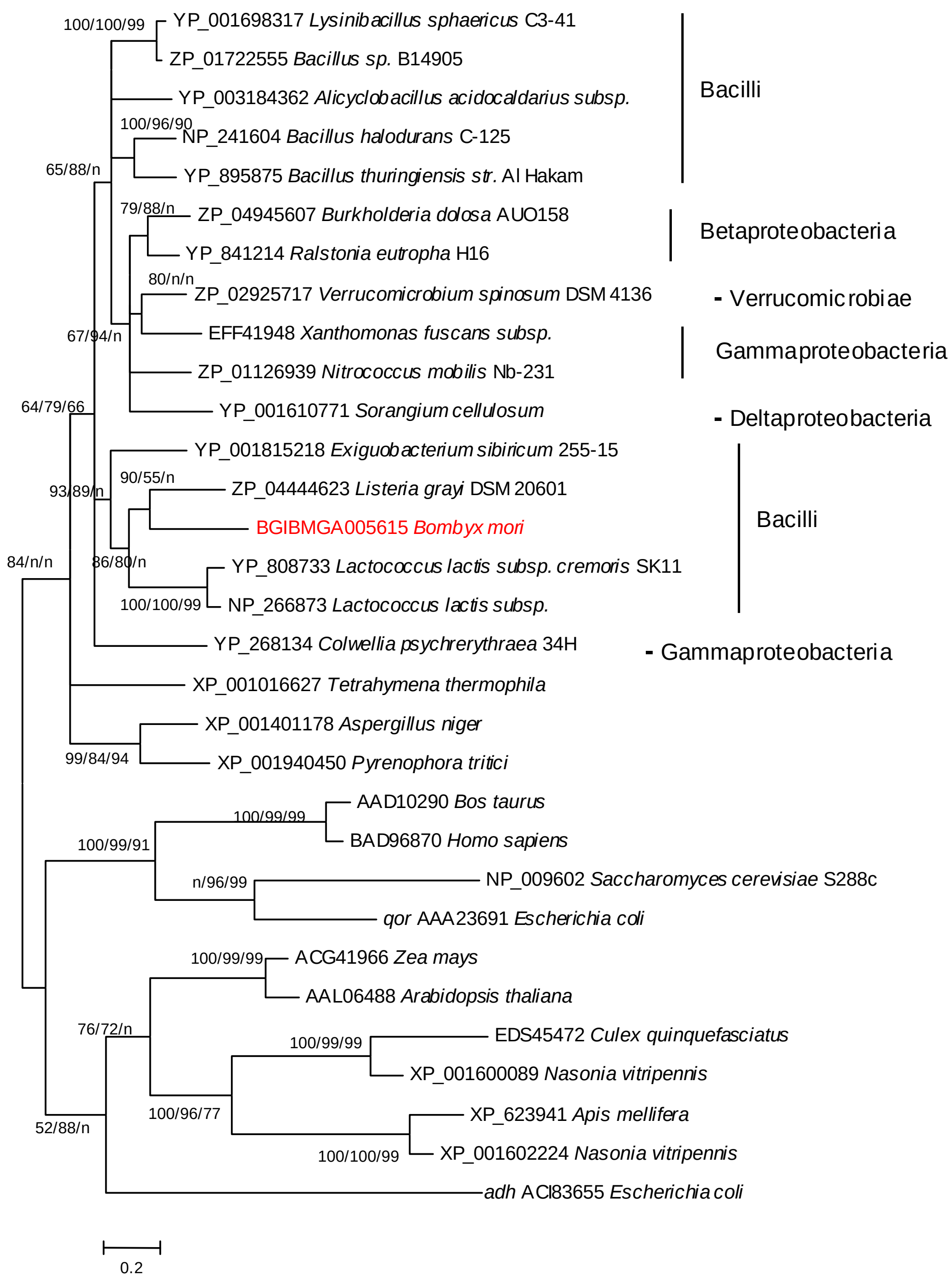

Figure S3 (a) Multiple alignment of amino acid sequences of BGIBMGA005615 and its homologs. Trigone represents conserved motif. Asterisk represents conserved active site in *E. coli qor* gene. (b) Phylogenetic tree of BGIBMGA005615 and its homologs. Numbers beside nodes indicate supporting values in methods of BI/ML/NJ.

Figure S4(a)

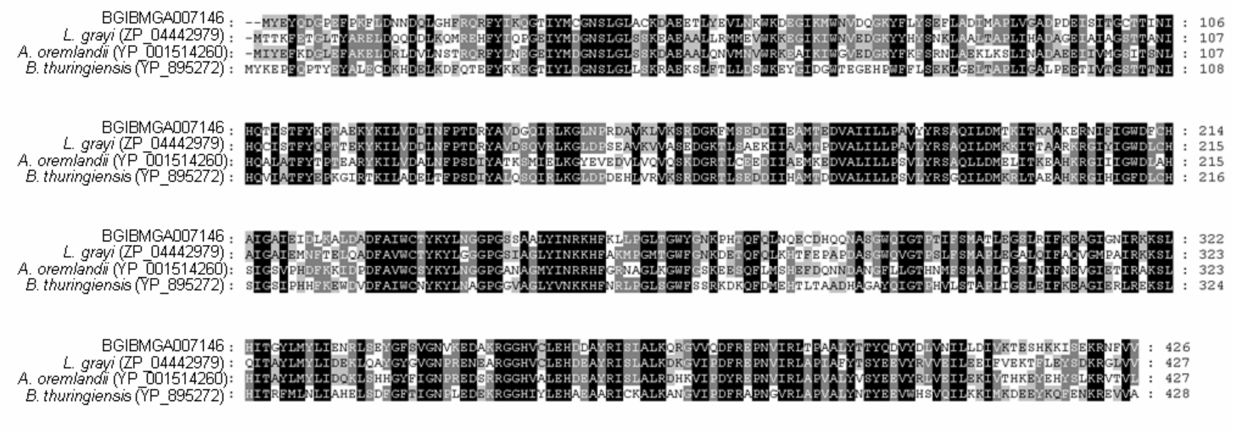

Figure S4(b)

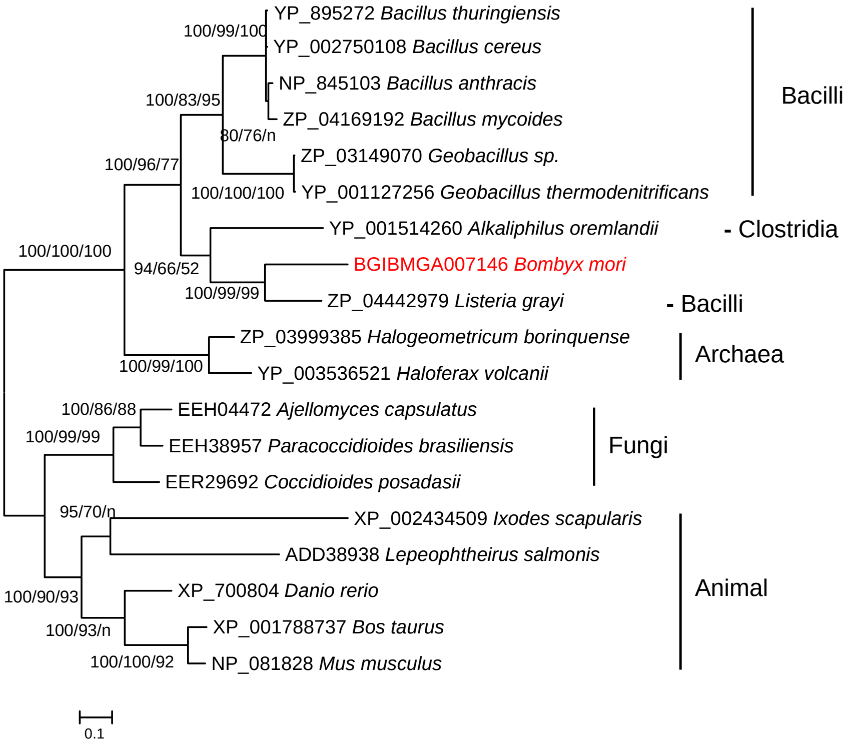

Figure S4 (a) Multiple alignment of amino acid sequences of BGIBMGA007146 and its homologs. (b) Phylogenetic tree of BGIBMGA007146 and its homologs. Numbers beside nodes indicate supporting values in methods of BI/ML/NJ.

Figure S5(a)

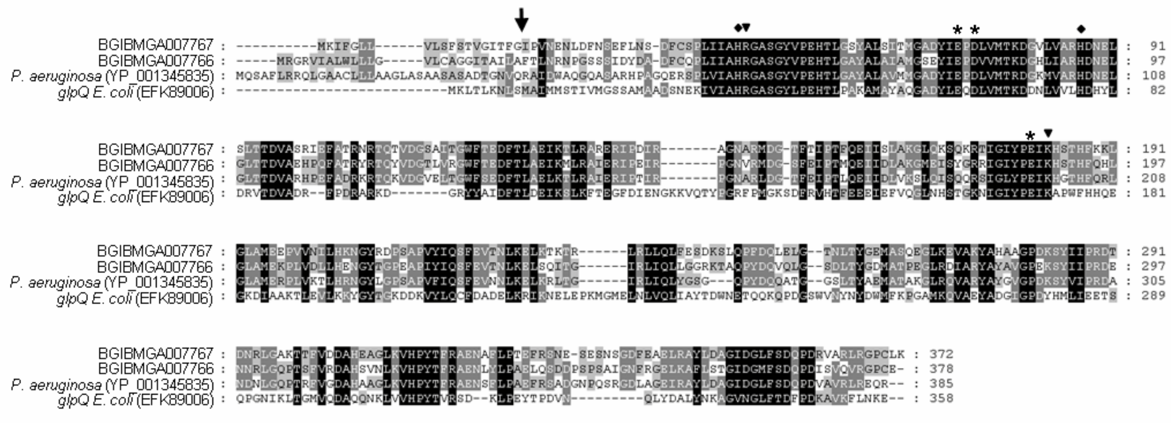

Figure S5(b)

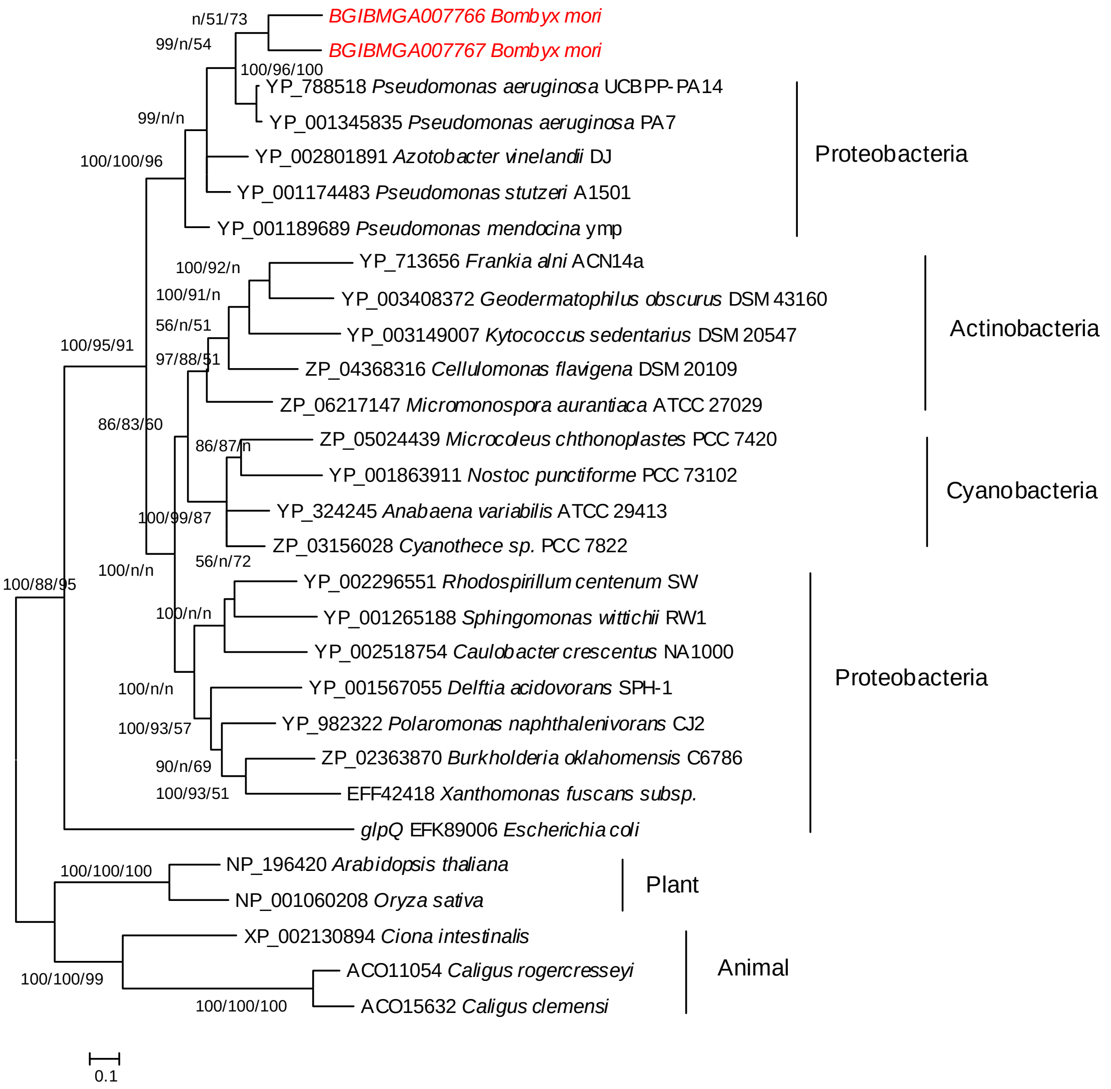

Figure S5 (a) Multiple alignment of amino acid sequences of BGIBMGA007766, BGIBMGA007767 and their homologs. Arrow represents the predicted cleavage site of signal peptide. Asterisk represents metal-binding site. Rhombus represents essential residue for catalysis. Trigone represents other conserved site. (b) Phylogenetic tree of BGIBMGA007766, BGIBMGA007767 and their homologs. Numbers beside nodes indicate supporting values in methods of BI/ML/NJ.

Figure S6(a)

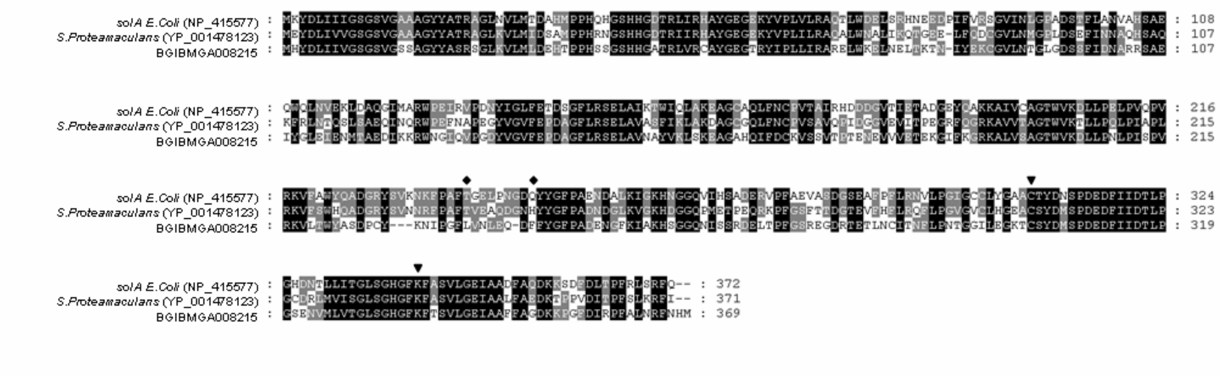

Figure S6(b)

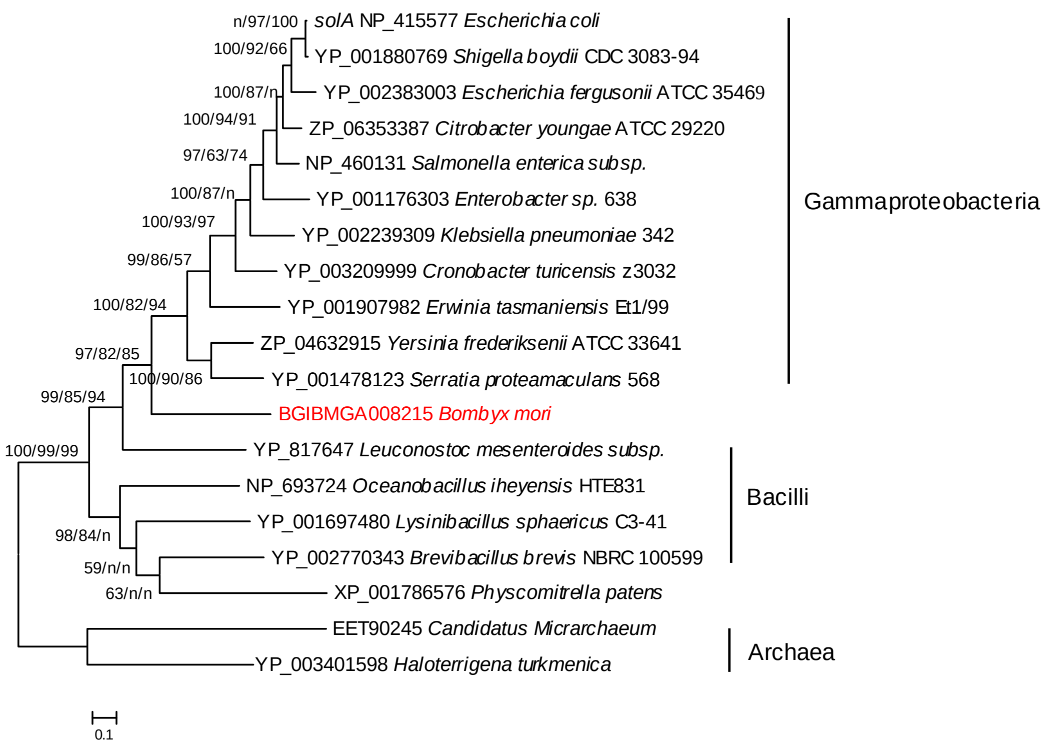

Figure S6 (a) Multiple alignment of amino acid sequences of BGIBMGA008215 and its homologs. Trigone represents conserved residue bonding to cofactor. Rhombus represents conserved catalysis site in *E. coli solA* gene. (b) Phylogenetic tree of BGIBMGA008215 and its homologs. Numbers beside nodes indicate supporting values in methods of BI/ML/NJ.

Figure S7(a)

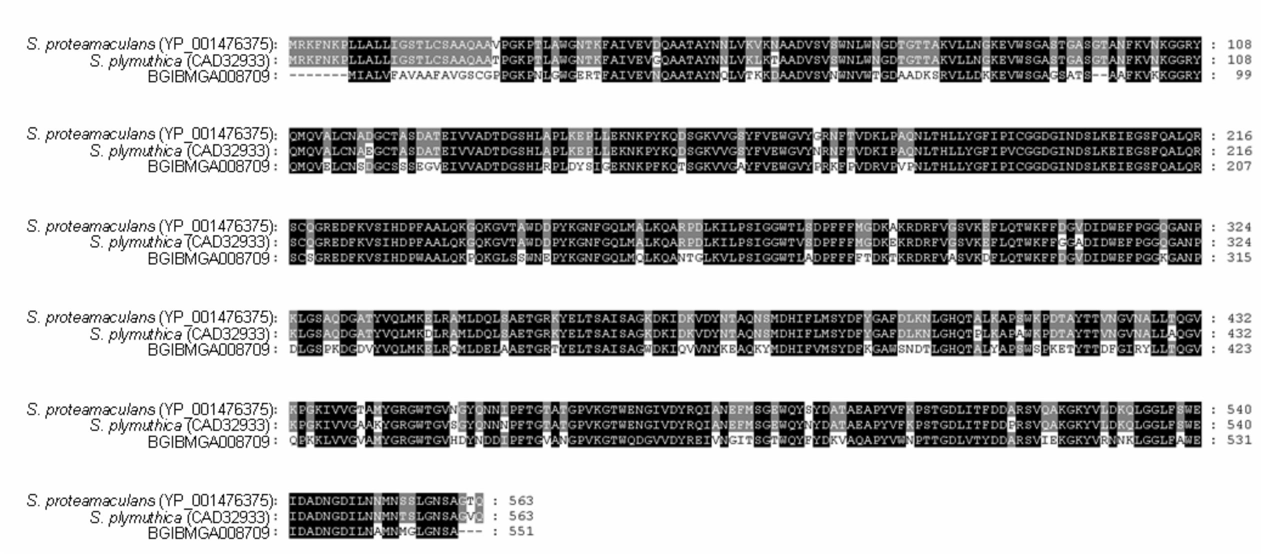

Figure S7(b)

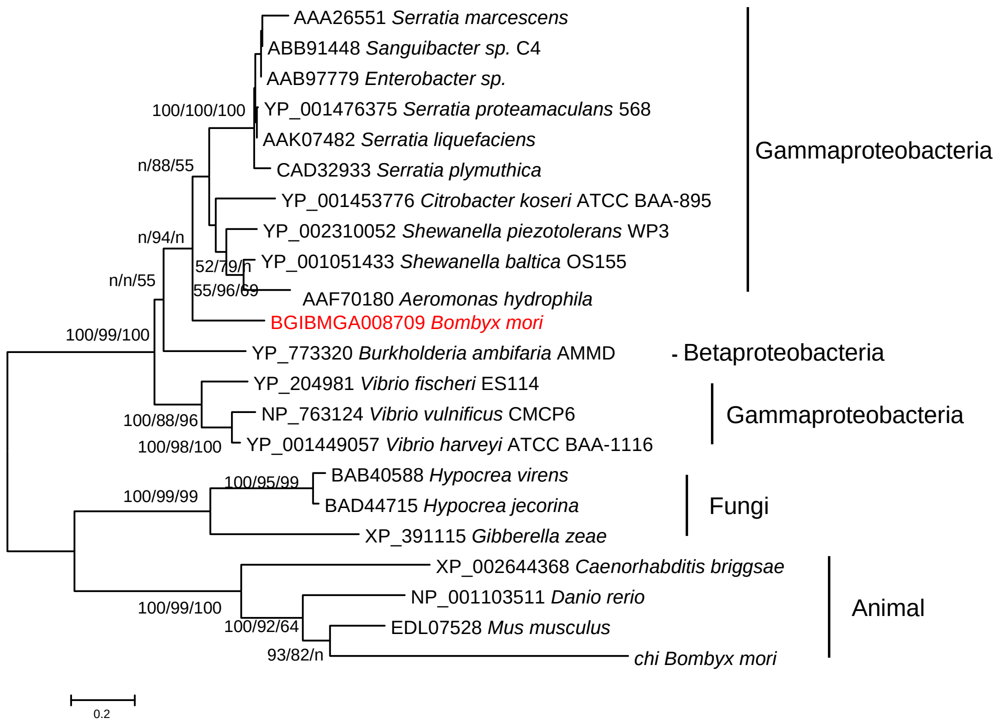

Figure S7 (a) Multiple alignment of amino acid sequences of BGIBMGA008709 and its homologs. (b) Phylogenetic tree of BGIBMGA008709 and its homologs. Numbers beside nodes indicate supporting values in methods of BI/ML/NJ.

Figure S8(a)

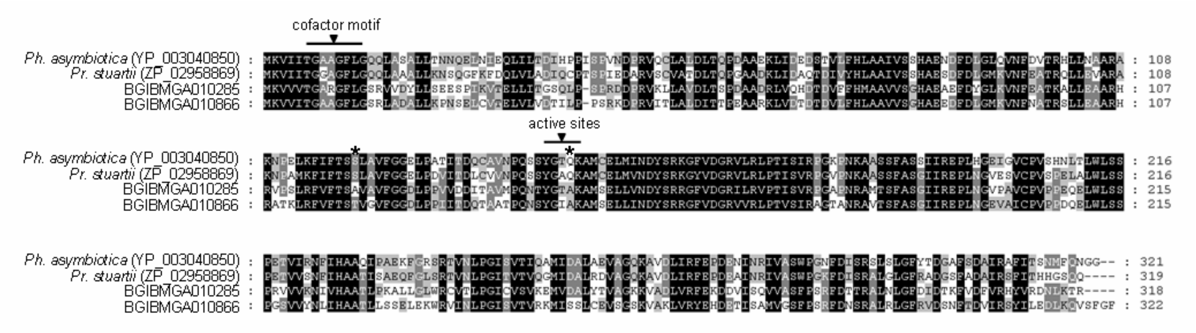

Figure S8(b)

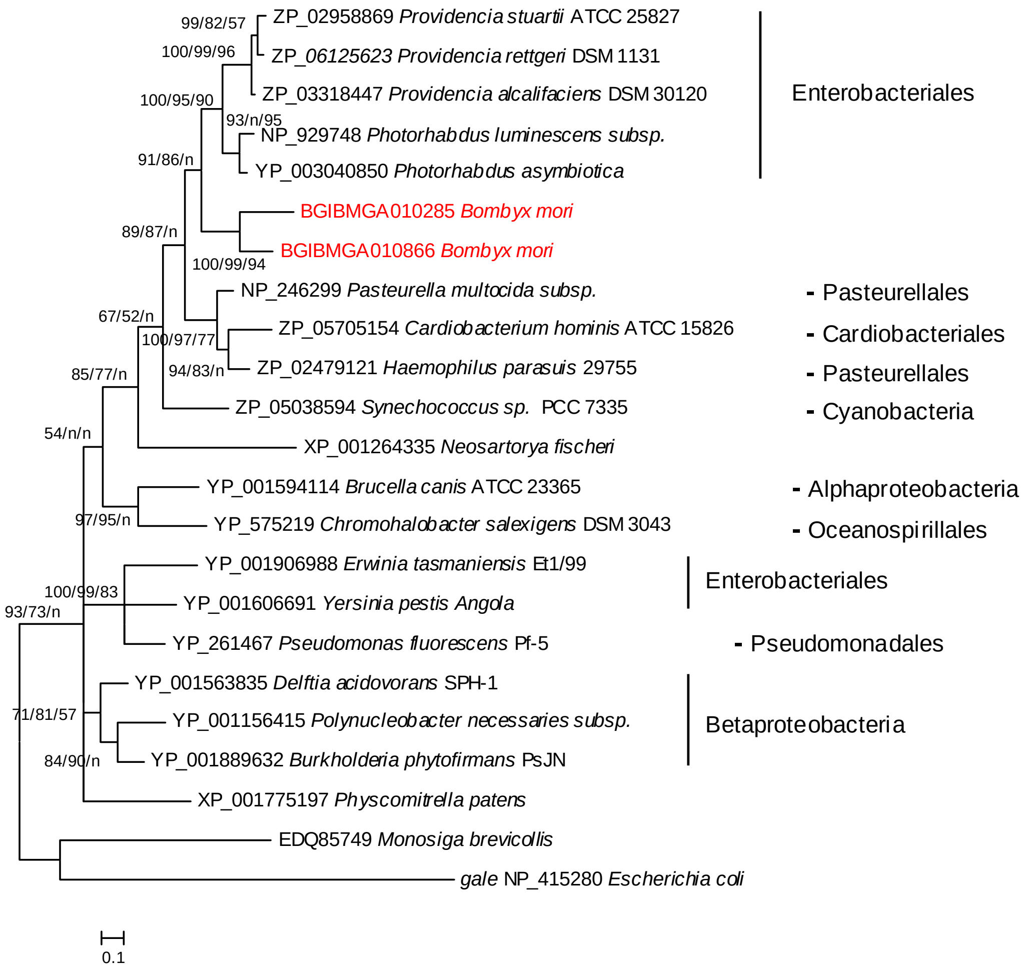

Figure S8 (a) Multiple alignment of amino acid sequences of BGIBMGA010285, BGIBMGA010866 and their homologs. Trigone represents conserved motif. Asterisk represents conserved active site in *E. coli gale* gene. (b) Phylogenetic tree of BGIBMGA010285, BGIBMGA010866 and their homologs. Numbers beside nodes indicate supporting values in methods of BI/ML/NJ.

Figure S9(a)

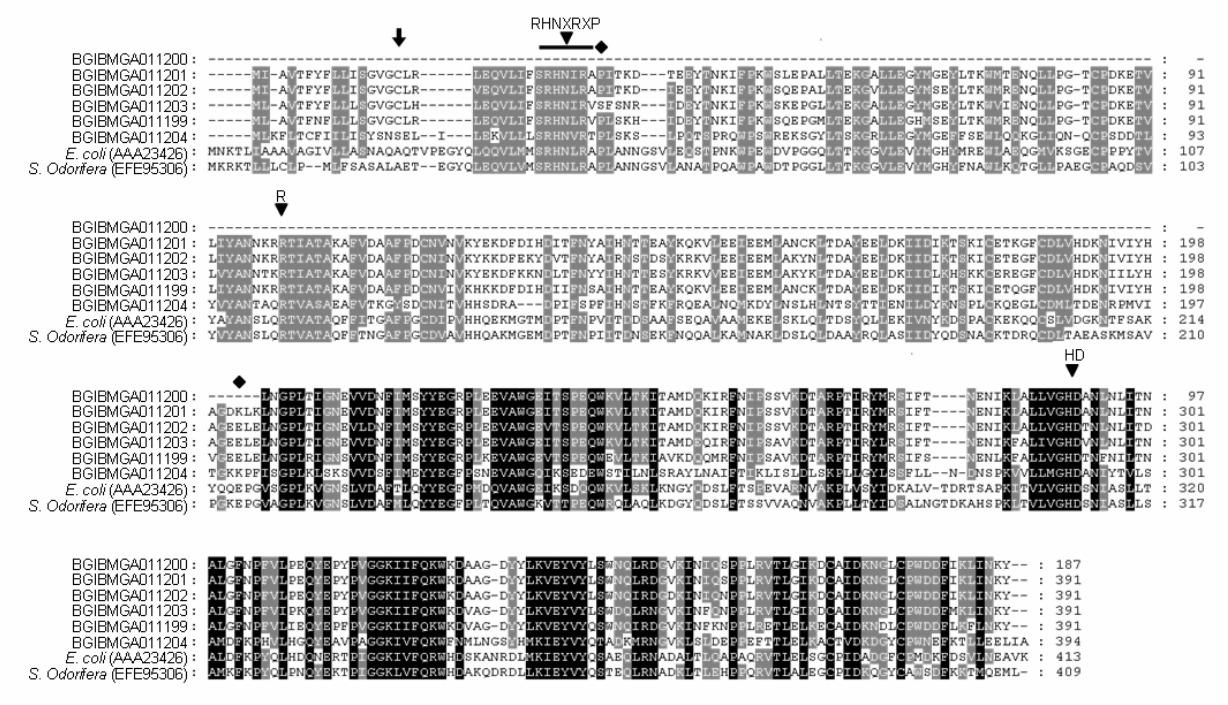

Figure S9(b)

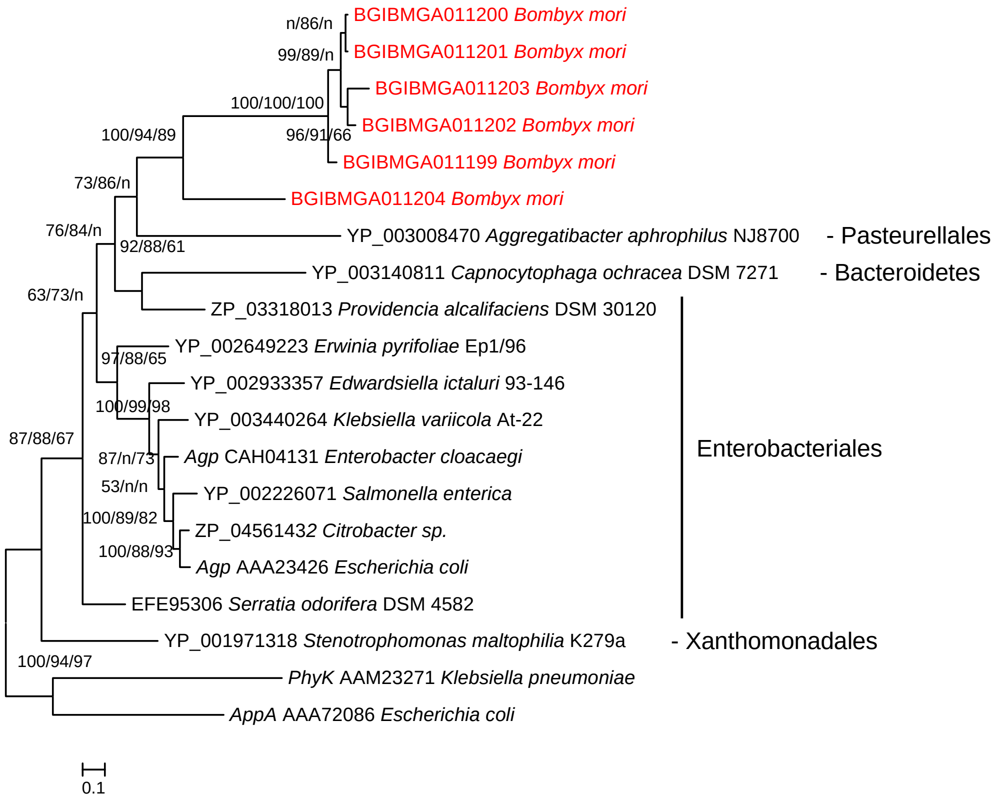

Figure S9 (a) Multiple alignment of amino acid sequences of BGIBMGA011199 type and their homologs. Arrow represents predicted cleavage site of signal peptide. Trigone represents the conserved motif of acid phosphatase. Rhombus represents conserved catalysis residues. (b) Phylogenetic tree of BGIBMGA011199 type of genes and their homologs. Numbers beside nodes indicate supporting values in methods of BI/ML/NJ.

Figure S10(a)

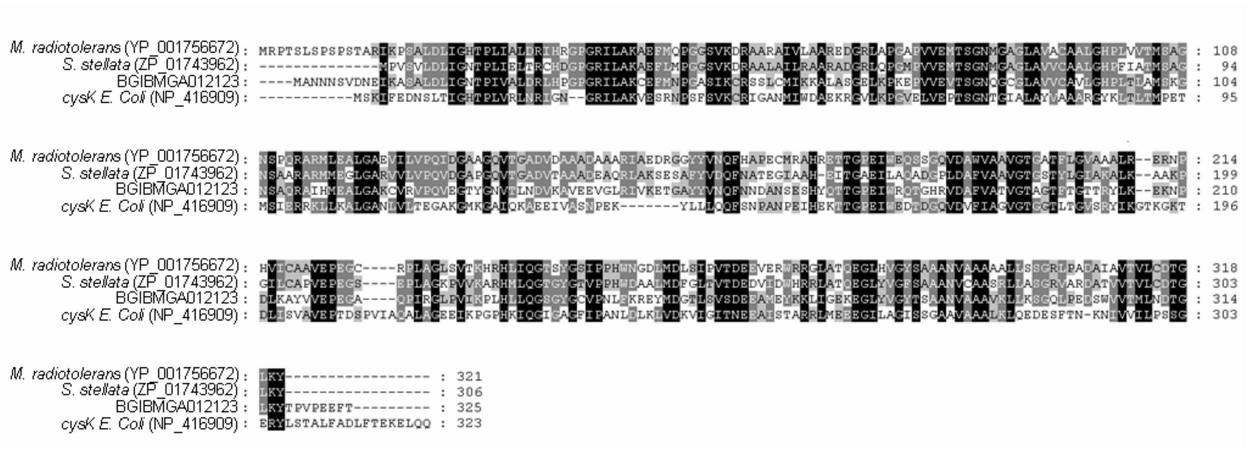

Figure S10(b)

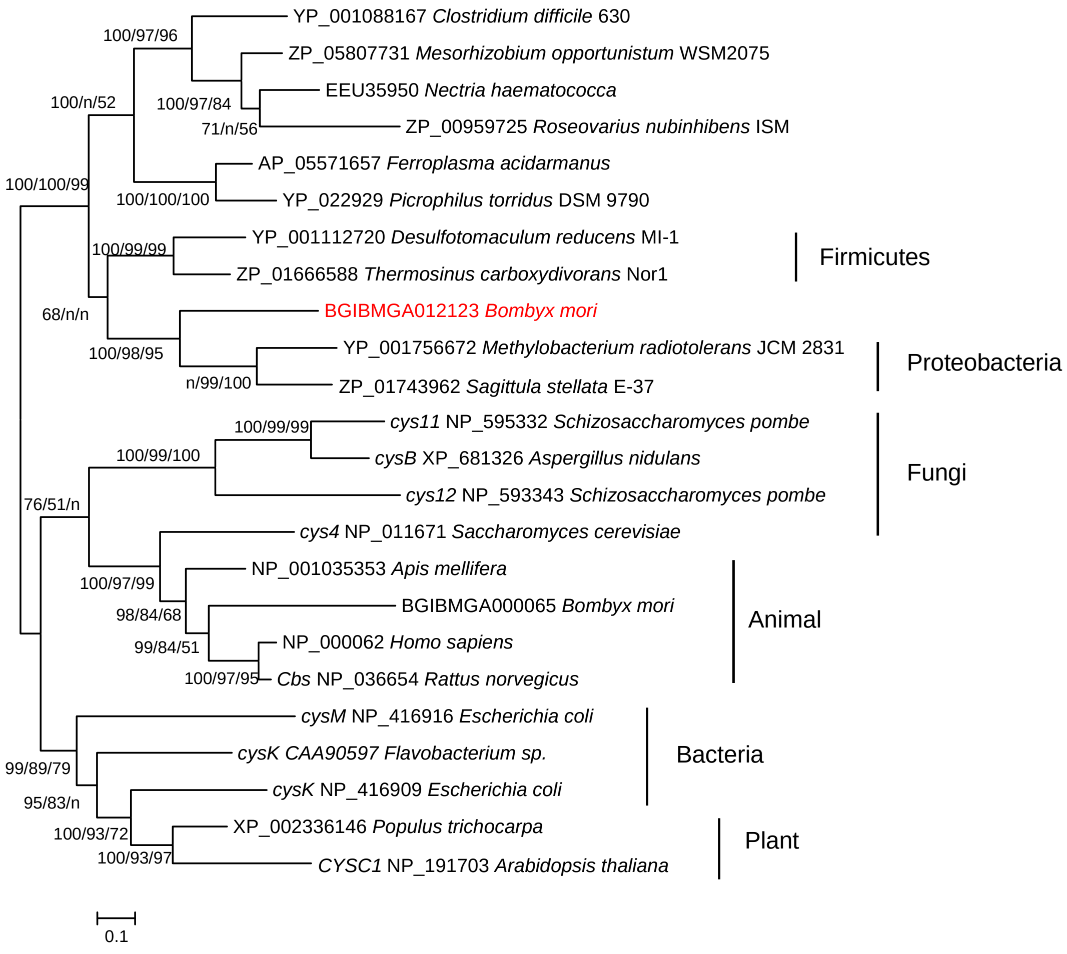

Figure S10 (a) Multiple alignment of amino acid sequences of BGIBMGA012123 and its homologs.

(b) Phylogenetic tree of BGIBMGA012123 and its homologs. Numbers beside nodes indicate supporting values in methods of BI/ML/NJ.

Figure S11(a)

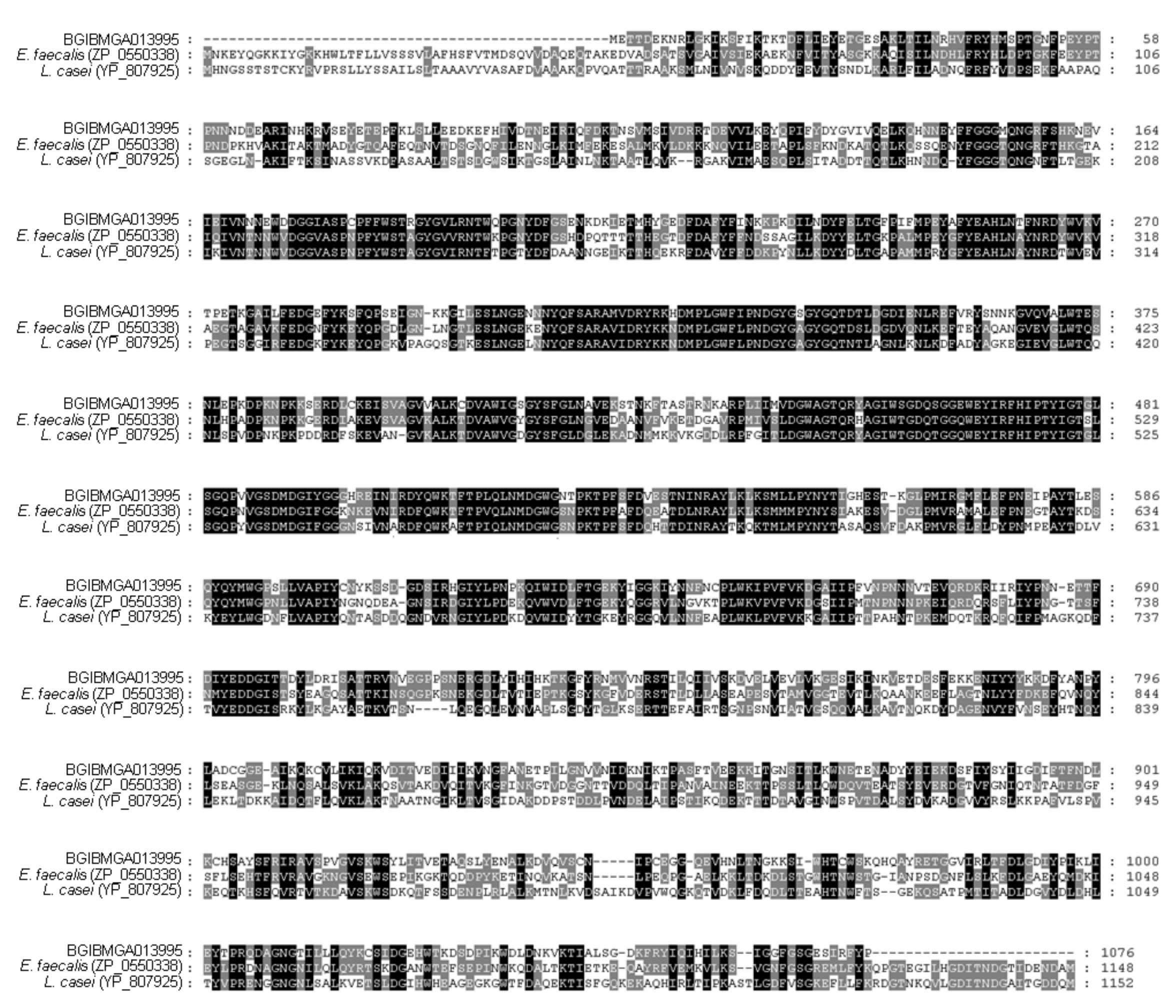

Figure S11(b)

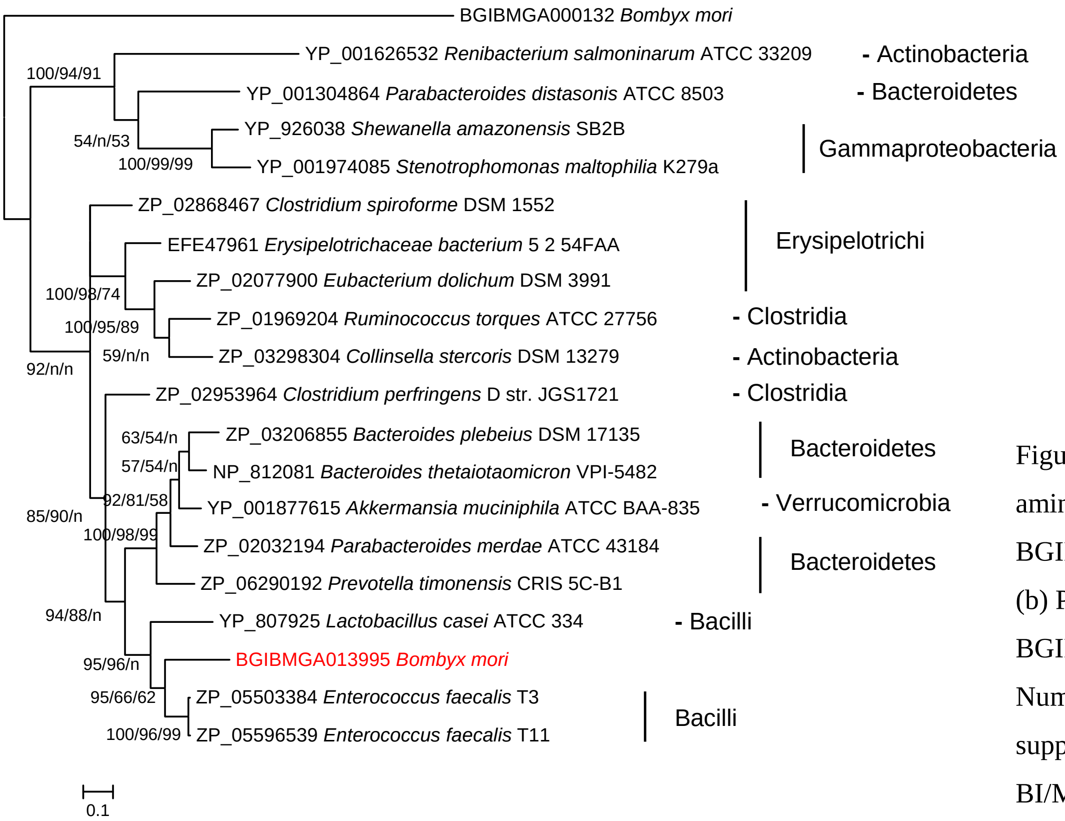

Figure S11 (a) Multiple alignment of amino acid sequences of BGIBMGA013995 and its homologs. (b) Phylogenetic tree of BGIBMGA013995 and its homologs. Numbers beside nodes indicate supporting values in methods of BI/ML/NJ.
