# Supplementary material for: Pathogen-origin horizontally transferred genes contribute to the evolution of Lepidopteran insects
Source: BMC Evol Biol. 2011 Dec 12;11:356. doi: 10.1186/1471-2148-11-356 (PMC3252269; doi:10.1186/1471-2148-11-356)
Supplement: Additional file 7 — Average similarity between detected HTGs and predicted donor sequences. Red curve indicates the normal distribution (mean, 41.03%; s.d., 2.62%) of sequence similarity based on random sampling results. Blue curve indicates the normal distribute (mean, 41.02%; s.d., 9.76%) of sequence similarity between 1176 silkworm genes and their bacterial homologs. Arrow indicates the position of mean value (50.36%) of 14 types of HTGs. [file 1471-2148-11-356-S7.PDF]

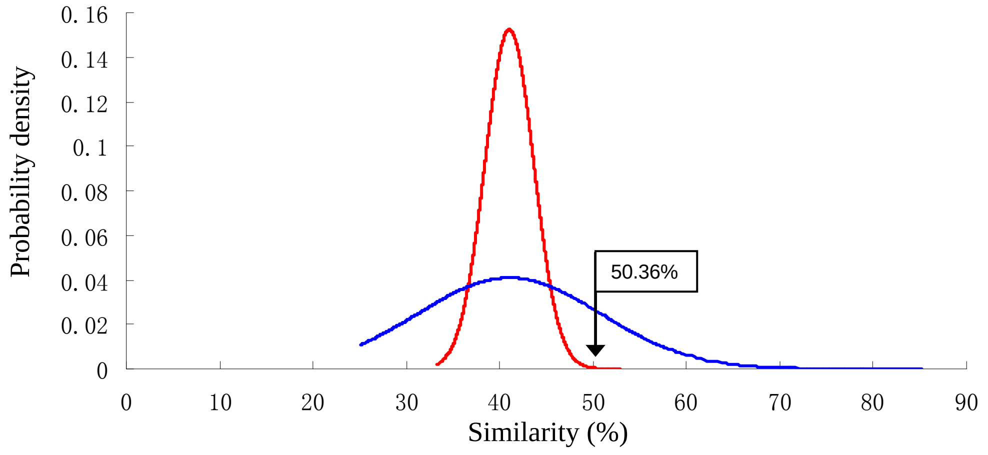

Additional file 7

Average similarity between detected HTGs and predicted donor sequences are significant larger. Red curve indicates the normal distribution (mean, 41.03%; s.d., 2.62%) of sequence similarity based on random sampling results. Blue curve indicates the normal distribute (mean, 41.02%; s.d., 9.76%) of sequence similarity between 1176 silkworm genes and their bacterial homologs. Arrow indicates the position of mean value (50.36%) of 14 types of HTGs.
