# Supplementary material for: Pathogen-origin horizontally transferred genes contribute to the evolution of Lepidopteran insects
Source: BMC Evol Biol. 2011 Dec 12;11:356. doi: 10.1186/1471-2148-11-356 (PMC3252269; doi:10.1186/1471-2148-11-356)
Supplement: Additional file 8 — GC content distribution of detected HTGs. The normal distribution (mean, 47.87%; s.d., 7.86%) indicates GC contents of the silkworm 14,623 genes. The open circles represent GC contents of silkworm HTGs (from left to right: BGIBMGA000070, BGIBMGA009498, mean value of BGIBMGA011199 type, BGIBMGA013995, BGIBMGA005615, BGIBMGA007146, BGIBMGA002521, mean value of BGIBMGA007766 type, BGIBMGA001284, mean value of BGIBMGA010285 type, BGIBMGA012123, mean value of BGIBMGA005555 type, BGIBMGA008215, BGIBMGA008709). The solid circles represent GC contents of corresponding donor sequences. The donors and recipients are connected with arrow lines. [file 1471-2148-11-356-S8.PDF]

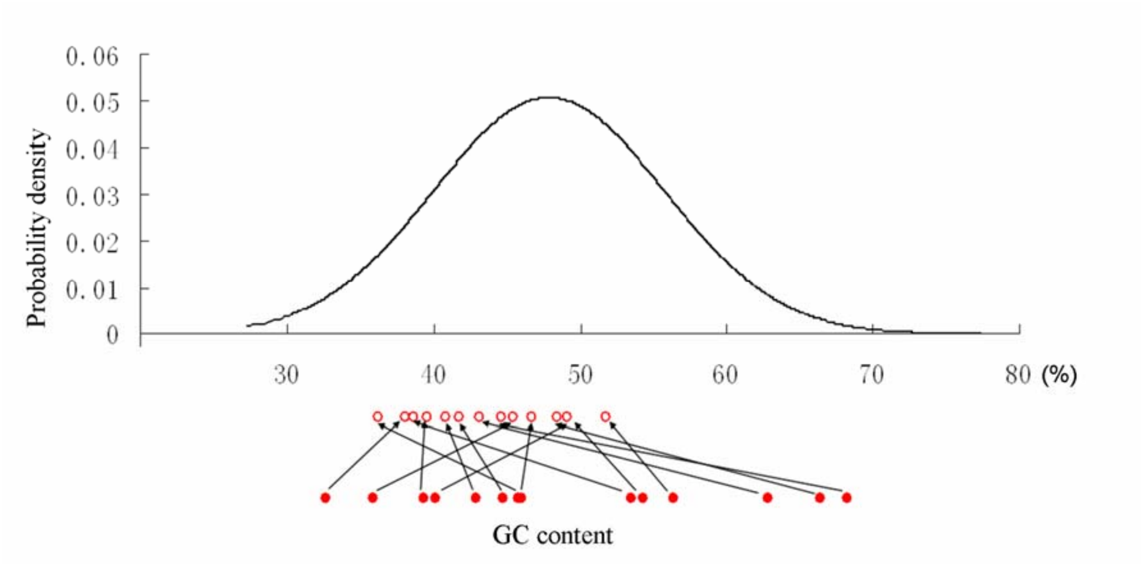

Additional file 8

GC content distribution of detected HTGs. The normal distribution (mean, 47.87%; s.d., 7.86%) indicates GC contents of the silkworm 14,623 genes. The open circles represent GC contents of silkworm HTGs (from left to right: BGIBMGA000070, BGIBMGA009498, mean value of BGIBMGA011199 type, BGIBMGA013995, BGIBMGA005615, BGIBMGA007146, BGIBMGA002521, mean value of BGIBMGA007766 type, BGIBMGA001284, mean value of BGIBMGA010285 type, BGIBMGA012123, mean value of BGIBMGA005555 type, BGIBMGA008215, BGIBMGA008709). The solid circles represent GC contents of corresponding donor sequences. The donors and recipients are connected with arrow lines.
